# Heat-related mortality trends under recent climate warming in Spain: A 36-year observational study

**DOI:** 10.1371/journal.pmed.1002617

**Published:** 2018-07-24

**Authors:** Hicham Achebak, Daniel Devolder, Joan Ballester

**Affiliations:** 1 Center for Demographic Studies (CED), Autonomous University of Barcelona (UAB), Barcelona, Spain; 2 Climate and Health Program (CLIMA), Barcelona Institute for Global Health (ISGlobal), Barcelona, Spain; University of Wisconsin, Madison, UNITED STATES

## Abstract

**Background:**

Anthropogenic greenhouse gas emissions have increased summer temperatures in Spain by nearly one degree Celsius on average between 1980 and 2015. However, little is known about the extent to which the association between heat and human mortality has been modified. We here investigate whether the observed warming has been associated with an upward trend in excess mortality attributable to heat or, on the contrary, a decrease in the vulnerability to heat has contributed to a reduction of the mortality burden.

**Methods and findings:**

We analysed a dataset from 47 major cities in Spain for the summer months between 1980 and 2015, which included daily temperatures and 554,491 deaths from circulatory and respiratory causes, by sex. We applied standard quasi-Poisson regression models, controlling for seasonality and long-term trends, and estimated the temporal variation in heat-related mortality with time-varying distributed lag nonlinear models (DLNMs). Results pointed to a reduction in the relative risks of cause-specific and cause-sex mortality across the whole range of summer temperatures. These reductions in turn explained the observed downward trends in heat-attributable deaths, with the only exceptions of respiratory diseases for women and both sexes together. The heat-attributable deaths were consistently higher in women than in men for both circulatory and respiratory causes. The main limitation of our study is that we were not able to account for air pollution in the models because of data unavailability.

**Conclusions:**

Despite the summer warming observed in Spain between 1980 and 2015, the decline in the vulnerability of the population has contributed to a general downward trend in overall heat-attributable mortality. This reduction occurred in parallel with a decline in the vulnerability difference between men and women for circulatory and cardiorespiratory mortality. Despite these advances, the risk of death remained high for respiratory diseases, and particularly in women.

## Abstract 2

Joan Ballester and colleagues reveal a general decline in heat-attributable deaths over the last 30 years in Spain, despite increasing temperatures, with the exception of respiratory disease, which is increasing, especially in women.

## Author summary

**Why was this study done?:**

- The Euro-Mediterranean region arises as a major climatic hot spot because of global warming.
- Warmer temperatures should in principle contribute to an increase in the number of deaths because of heat.
- We do not know yet if and to what extent societal adaptation and/or socioeconomic development is modifying this expected increase.

**What did the researchers do and find?:**

- We analysed daily mortality records from 47 major cities in Spain.
- There has been a general and sustained decline in the vulnerability of the population since 1980.
- Despite the observed warming, the decline of the vulnerability has generally contributed to a progressive reduction in the number of deaths attributed to heat since 1980.

**What do these findings mean?:**

- It is generally believed that climate change will cause an increase in heat-related mortality.
- Societal adaptation and/or socioeconomic development contributed, up to now, to a general decline in heat-related mortality.
- It is still uncertain if this decline in heat-related mortality will also occur at higher future levels of climate warming.

## Introduction

Anthropogenic climate change represents a major threat for human health and a challenge for public health services [1]. One of its most important effects is the potential increase in heat-related mortality resulting from rising temperatures and the associated increase in the frequency, intensity, and duration of extreme heat events [2,3]. However, the extent of this impact will not only depend on the increase in the level of exposure to heat but also on any underlying change in the vulnerability of the exposed population [4].

Several factors have the potential to modify population vulnerability over time and, therefore, the eventual incidence of increasing temperatures on heat-related mortality. In ageing societies such as Europe, the rising elderly population is expected to increase vulnerability to high ambient temperatures, given that the elderly have diminished physiological capacity for the regulation of body core temperature under heat stress conditions [5]. On the contrary, general improvements in housing conditions (e.g., wider use of air conditioning systems in retirement homes) and healthcare services (e.g., improved treatment of heat-related morbidity) [6,7] as well as some planned adaptive measures to reduce the exposure and vulnerability to heat (e.g., implementation of effective heat health warning systems) [8] could all contribute to reducing the negative health consequences of temperatures and warming trends.

The Euro-Mediterranean region arises as a major climatic hot spot as a result of global warming [9], and, particularly, Spain was the country with the largest relative number of excess deaths during the record-breaking summer 2003 heat wave [10], even if interannual temperature anomalies were twice as large in France during the episode [11]. Some authors have shown that there has been a decline in heat-mortality associations during the last decades in some, albeit not all, of the countries [12–15], and especially since this event in a subset of locations [16–18]. In the case of Spain, the vulnerability associated with heat was found to decrease for the range of extreme summer temperatures [12], suggesting an adjustment response of the Spanish society to rising temperatures despite the ageing of the population. However, evidence about the risk attributable to heat, either as absolute or relative excesses or its temporal evolution, is lacking, with no clear upward or downward trend in the number of deaths. Furthermore, changes by cause of death and sex have not been described for Spain, nor for the rest of the countries.

In the present work, we assess the impact of the summer warming observed in Spain during the period 1980–2015 on cause-specific mortality by sex. The main objective was to determine whether warmer summers were associated with an upward trend in excess mortality attributable to heat. Addressing the early impacts of increased ambient temperatures on human health is a relevant question that could translate into more effective public health adaptation strategies to current and future climate change conditions.

## Methods

### Study design and data

The Spanish National Statistics Institute (INE) provided daily death counts from circulatory (ICD-9: 390–459, ICD-10: I00–I99) and respiratory (ICD-9: 460–519, ICD-10: J00–J99) diseases, disaggregated by sex and covering the period from 1 January 1980 to 31 December 2015 in 47 major Spanish cities. Daily mortality data had no missing value. We derived daily mean 2-meter temperature observations from the European Climate Assessment and Dataset (ECA&D), which were computed as the average between daily maximum and minimum values from meteorological stations. About 1% of the temperature time series was missing data.

### Statistical analysis

The analysis was restricted to the warm season from June to September, and it was performed in two stages. In the first part, standard quasi-Poisson regression models allowing for overdispersion were individually applied in each of the 47 cities included in the analysis in order to estimate location-specific temperature-mortality associations, summarised in terms of relative risk (RR) values by cause of death and sex. The seasonal trend was controlled for in the models by using a natural cubic B-spline of day of the season, with 2 degrees of freedom per year/summer and equally spaced knots, and it was allowed to vary from one year to another through the specification of an interaction between the B-spline and indicators of summer/year. The models also included a natural cubic B-spline of time, with 1 degree of freedom per decade and equally spaced knots to control for the long-term trend, as well as a categorical variable to control for the day of the week.

The complex nonlinear and delayed dependencies found for temperature and mortality were captured by using a distributed lag nonlinear model (DLNM), a flexible methodological framework widely used to investigate the health effects of air pollution and temperature. This model is based on the definition of a cross-basis function, obtained by the combination of two functions describing the exposure-response association and the lag-response association [19]. Specifically, the exposure-response curve was modelled through a natural cubic B-spline, with one internal knot placed at the 15th percentile of the daily temperature distribution, and the lag-response curve was modelled through a natural cubic B-spline, with an intercept and two internal knots placed at equally spaced values in the log scale, with a lag period extended up to 10 days to account for the lagged effects of heat and short-term harvesting. In this way, the overall effect of a given summer day temperature on mortality was defined as the sum of the effect on that day and the 10 subsequent days. These modelling choices were thoroughly tested in sensitivity analyses, which are shown in the Supporting information (S1 Fig). The Poisson regression model for the whole study period was given as follows:

$$Log\;E\;(Y_{t})=intercept+cb+dow+S1\left(dos,\;df=2\right):factor\left(year\right)+S2\left(time,\;df=1\;per\;decade\right)$$

where Y_t_ denotes the series of daily mortality counts, cb the cross-basis matrix produced by DLNM, dow the day of the week, S1 the natural cubic B-spline of the day of the season, and S2 the natural cubic B-spline of time.

To model temporal variations in the bidimensional exposure-lag-response associations between temperature and mortality, the DLNM model described above was extended to a time-varying DLNM, specified through a linear interaction between the cross-basis and time variables [20]. This extension allowed us to predict the temperature-mortality relationship for each summer in the series, by centring the time variable in the central day of the respective summer season.

$$Log\;E\;(Y_{t})=intercept+cb+dow+S1\left(dos,\;df=2\right):factor\left(year\right)+S2\left(time,\;df=1\;per\;decade\right)+int\left(time*cb\right)$$

In the second stage, a simple multivariate random-effects meta-analysis [21] was used to estimate the average temperature-mortality association across cities for the whole study period (estimates provided by the model without interaction) and for each year in the series (estimates provided by the model with interaction). The fitted meta-analytical model was also used to derive the best linear unbiased predictions (BLUPs) of the temperature-mortality relationships and the related point of minimum mortality temperature (MMT) in each location. Uncertainty in estimated MMTs was investigated through the method described in Tobías et al. [22].

To assess the temporal evolution in the effect of heat on mortality, the pooled RR curves from the time-varying DLNMs with interaction terms were compared between years. The temporal variations in the RR curves were analysed by means of a multivariate Wald test on the pooled coefficients of the interaction terms, which represent the change in the average temperature mortality curves. The null hypothesis of the test is that no change in the temperature-mortality association ocurred throughout the study period. We also summarised the results by calculating the pooled RR at the 90th and 99th temperature percentiles from year-specific curves between 1980 and 2015.

The mortality burden attributable to heat across the whole study period, reported as relative excess (i.e., attributable fraction) of deaths, was estimated by using the methodology developed by Gasparrini and Leone [23]. First, the RR of mortality corresponding to each day and city was used to calculate the attributable fraction of deaths on that day and the next 10 days. Then, the daily attributable number of deaths was computed by multiplying the daily attributable fraction by the daily number of deaths. The overall number of attributable deaths caused by heat was given by the sum of the contributions from all days of the series with temperatures higher than the value of MMT derived from the BLUP of the model with no interaction in each city, and its ratio with the total number of deaths provided the total heat-attributable fraction. This attributable component was separated further into the contributions of moderate and extreme heat. Moderate heat was defined as the range of temperatures between the city-specific MMT and the city-specific 97.5th daily temperature percentile, and extreme heat as the range of temperatures warmer than this threshold. We also computed the attributable risk of heat for each summer from time-varying DLNMs (model with interaction) to investigate temporal variations in heat-attributable deaths. Confidence intervals (CIs) of attributable risk were obtained empirically through Monte Carlo simulations.

All statistical analyses were performed with R software (version 3.4.3) using functions from the packages *dlnm* (first-stage regression) and *mvmeta* (second-stage meta-analysis).

No data-driven changes were done during the analyses. The development of the statistical analysis plan, including changes inspired by referees, is described in S1 Analysis Plan. This study is reported as per the Strengthening the Reporting of Observational Studies in Epidemiology (STROBE) guidelines (S1 STROBE Checklist).

## Results

**Fig 1:**
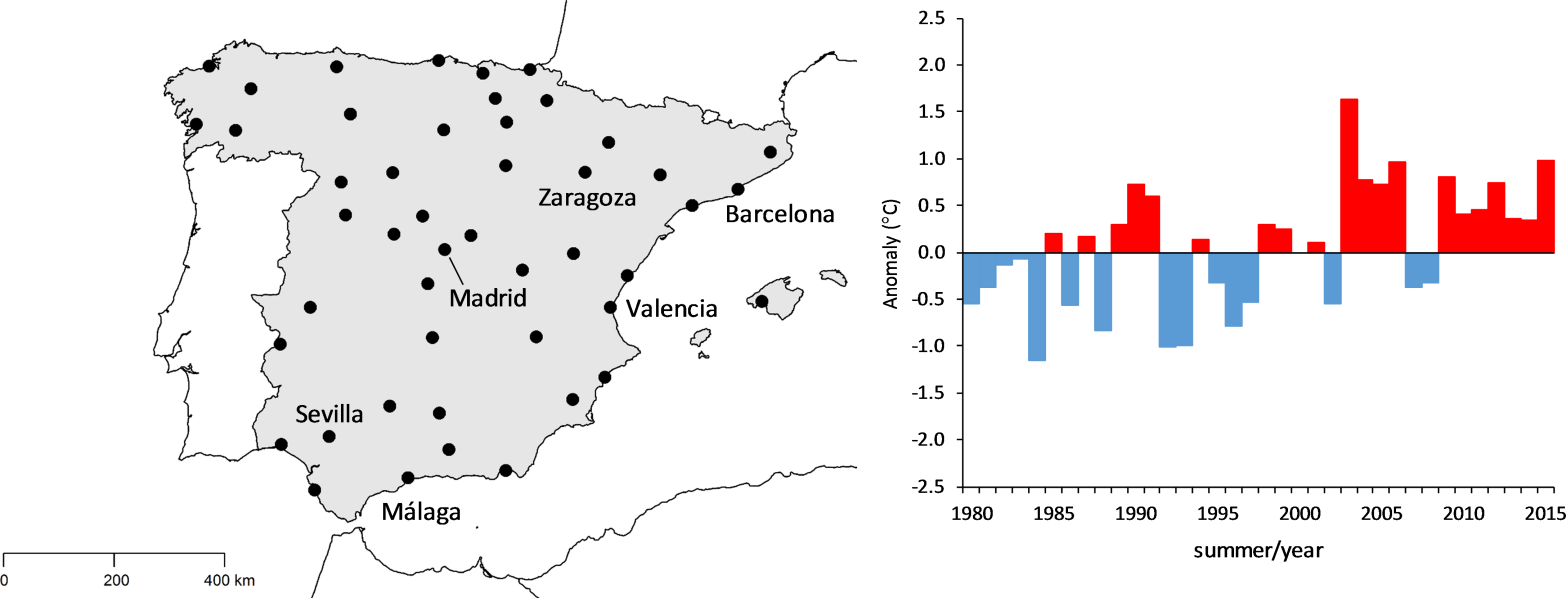
Geographic distribution of the 47 major Spanish cities (left) and the corresponding evolution of overall summer mean temperature (expressed as the difference relative to the average of the 1981–2010 period, right).

We analysed data from 47 major cities representing about 32% of the total Spanish population, which includes 544,491 summer deaths, corresponding to the period 1980–2015. Circulatory counts represented 78.9% of the total cardiorespiratory mortality (here the word ‘cardiorespiratory’ is used to refer to deaths from circulatory and respiratory deaths together), while respiratory deaths accounted for the remaining 21.1%. The temporal pattern of each cause of death was similar in men and women (S2 Fig), with a decline in the number of deaths from circulatory diseases and an increase in the number of deaths from respiratory diseases. Nevertheless, mortality decreased at a slower pace in women for circulatory diseases, therefore increasing the magnitude of the difference between women and men. Moreover, for respiratory diseases, mortality increased at a faster pace in women, therefore reducing the magnitude of the difference between women and men. The geographic distribution of the cities included in the analysis, along with the corresponding evolution in overall summer mean temperature, are displayed in Fig 1. As expected, summer temperatures have been increasing, on average, at a rate of 0.32°C per decade.

**Fig 2:**
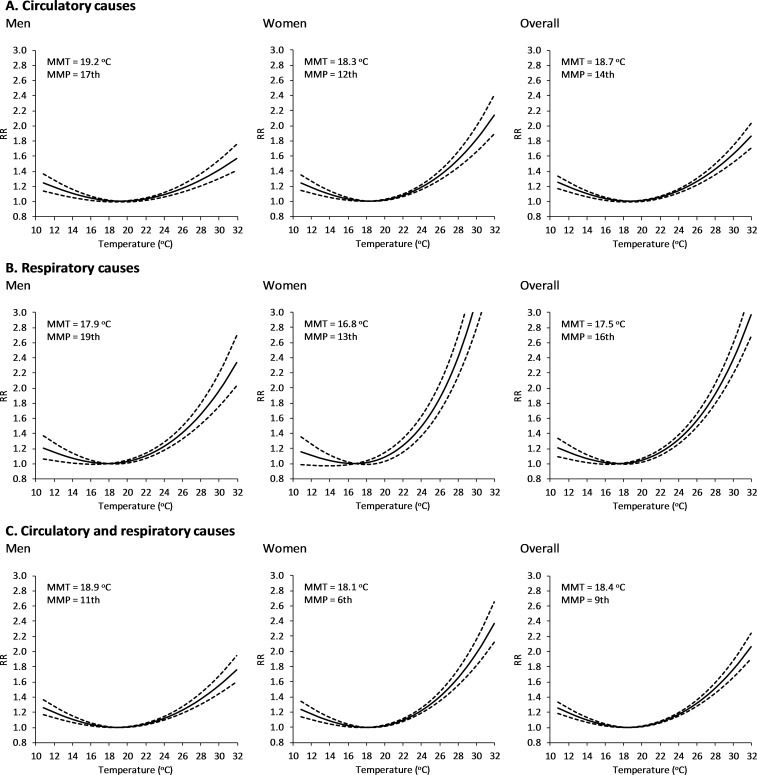
Temperature-mortality relationships from the model with no interaction for the period 1980–2015. The dashed lines represent the 95% empirical confidence interval. MMP, minimum mortality percentile; MMT, minimum mortality temperature; RR, relative risk.

Fig 2 depicts the pooled RR values associated with the temperature-mortality relationships by cause of death and sex from the model with no interaction, interpreted as the average relation throughout the whole study period (1980–2015). The temperature-mortality relationships computed as BLUPs for the 47 cities and the corresponding MMTs are provided in the Supporting information, S3 Fig and S4 Fig. All these curves are J shaped, indicating a monotonically increasing mortality risk for temperatures above and below the MMT. The slope of the curve above this point varied greatly among cases, being generally larger for respiratory than circulatory diseases, and for women than for men. Thus, in each of these two groups of causes, taken either individually or together, women showed systematically higher values of RR for the whole range of warm temperatures, with generally lower MMT than men. For the ensemble of cities, the values of the MMT and the summer mean temperature are largely correlated, with a spatial dependency of between 0.81 and 0.96°C in MMT per 1°C in summer mean temperature (Student *t* test/*p*-value < 0.001, S5 Fig).

**Fig 3:**
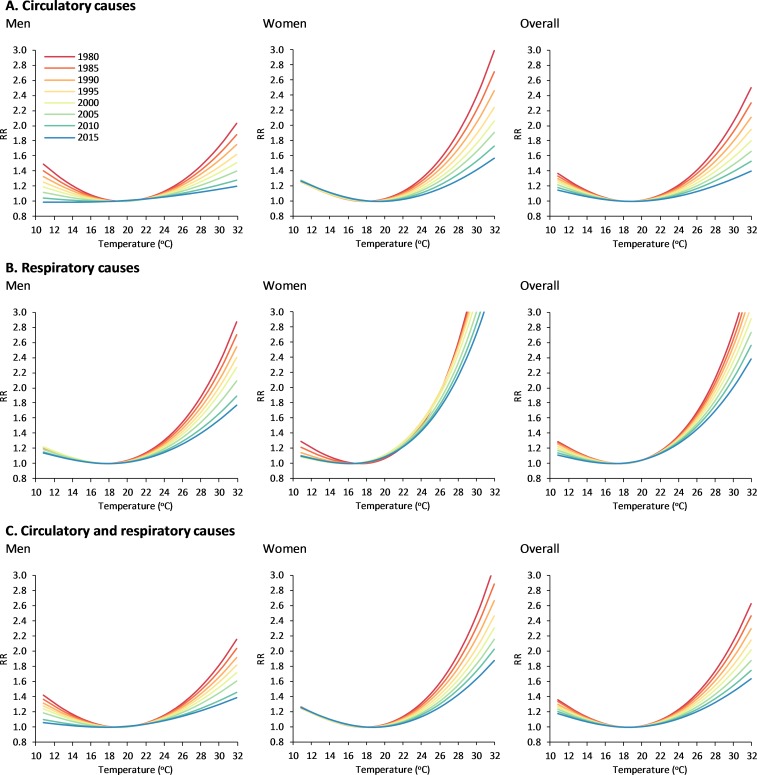
Temperature-mortality relationships from the model with interaction (time-varying DLNM) for representative years. DLNM, distributed lag nonlinear model; RR, relative risk.

**Fig 4:**
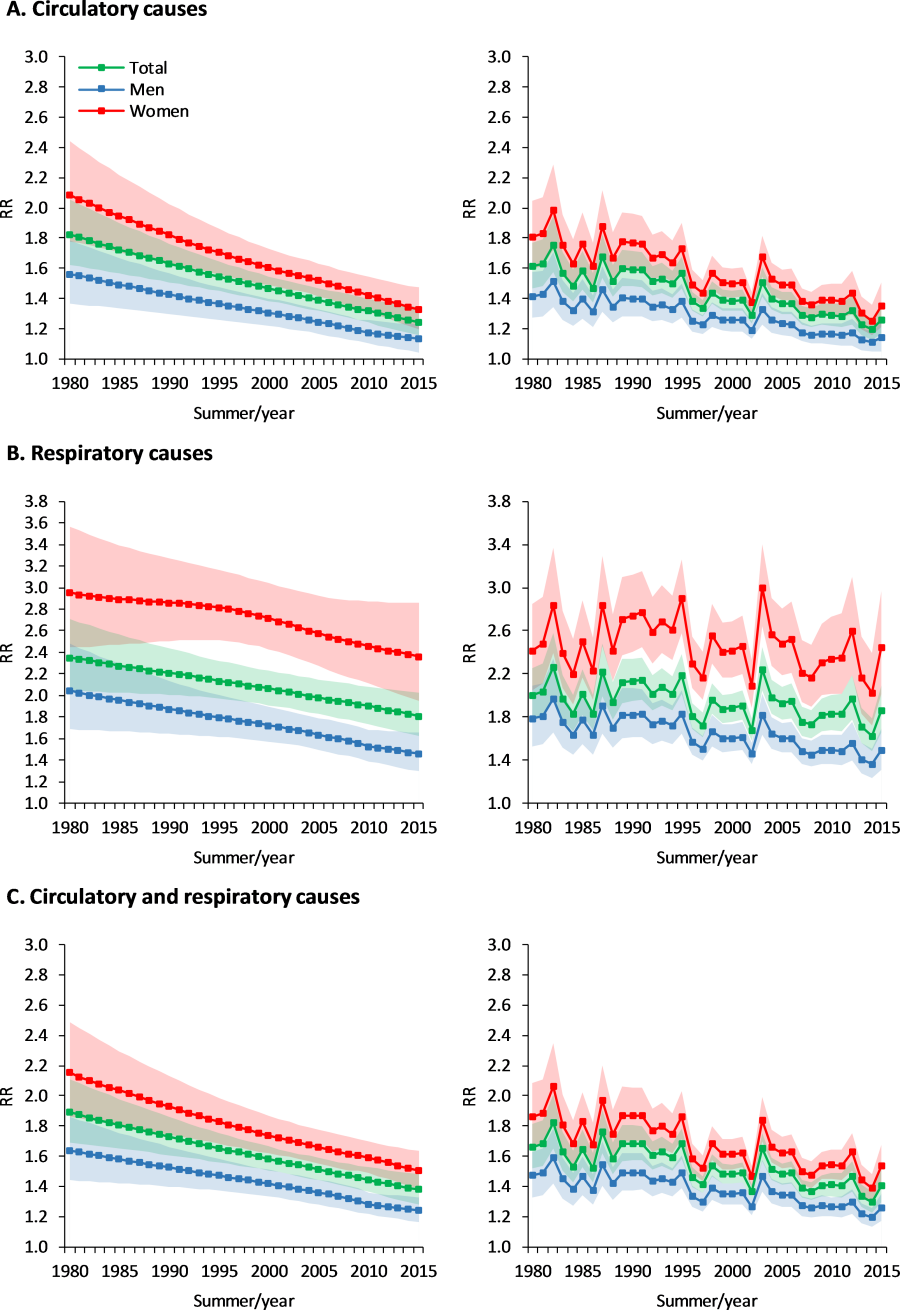
Temporal evolution of mortality RR at the 99th temperature percentile from the model with interaction (time-varying DLNM). In the left column panels, RR estimates correspond to the 99th temperature percentile of the summer time series for the whole study period, while in the right column graphs, they correspond to the 99th temperature percentile of the summer days of the given year (i.e., the 99th percentile of the 122 daily summer values of the year, computed separately for each year). The shaded areas represent the 95% empirical confidence interval. DLNM, distributed lag nonlinear model; RR, relative risk.

Results from the analysis of the temporal evolution of the temperature-mortality associations predicted from the model with interaction (time-varying DLNM) are summarised by cause of death and sex in Figs 3 and 4. On the one hand, Fig 3 displays the comparison of the pooled RR curves for representative years (i.e., every 5 years; see also S6 Fig for estimates in each of the 47 cities for years 1980 and 2015). On the other hand, Fig 4 shows the temporal evolution of the pooled RR corresponding to the 99th temperature percentile of the whole time period (left panels) and corresponding to the time-varying 99th temperature percentile computed from data of each individual summer (right panels; find equivalent results for the 90th percentile in S7 Fig). Temporal changes suggest a strong and progressive reduction in the RR of heat-related mortality during the whole study period for the pooled analysis and generally for the ensemble of cities, but despite these advances, the RR to extreme warm temperatures remained high for respiratory diseases, and particularly in women. The significance test (S1 Table) indicates strong evidence for significant temporal changes in the RR curves, with the only exception being respiratory mortality for women. As a result of all these results, differences in mortality risk between men and women from both circulatory and cardiorespiratory diseases generally declined during the study period.

Table 1 summarises the estimates of mortality deaths attributable to heat during the whole study period. The overall fraction of deaths caused by summer heat was 10.65% (95% CI 9.93–11.33), with moderate heat being responsible for almost nine times as many deaths as extreme heat. This was explained by the fact that the days with moderate temperatures occur more frequently in the series, and, therefore, they have a higher absolute impact on the total number of deaths. The attributable fraction due to respiratory causes (17.60%, 95% CI 16.00–18.95) was twice as high as that from circulatory causes (8.94%, 95% CI 8.22–9.61), although in absolute terms circulatory deaths accounted for most of the mortality burden. When considering these two groups of causes by sex, both the attributable fractions and numbers were substantially higher for women. The same pattern was separately observed for moderate and extreme heat.

**Table 1:** Mortality attributable fraction and number because of moderate and/or extreme heat, together with the 95% empirical confidence interval. Attributable fractions and numbers were predicted from the model without interaction and, therefore, are interpreted as values representing the whole study period (1980–2015).

**A. Attributable fraction of deaths**							
		**Heat**		**Moderate heat**		**Extreme heat**	
		**AF**	**95% CI**	**AF**	**95% CI**	**AF**	**95% CI**
Circulatory causes		8.94%	(8.22–9.61)	8.00%	(7.34–8.62)	0.94%	(0.87–0.99)
	Men	5.60%	(4.62–6.50)	4.96%	(4.07–5.79)	0.64%	(0.55–0.71)
	Women	11.71%	(10.75–12.64)	10.54%	(9.66–11.41)	1.17%	(1.09–1.23)
Respiratory causes		17.60%	(16.00–18.95)	15.88%	(14.36–17.17)	1.73%	(1.64–1.79)
	Men	13.80%	(12.28–15.20)	12.44%	(11.05–13.76)	1.35%	(1.23–1.44)
	Women	24.26%	(20.75–26.57)	22.02%	(18.63–24.26)	2.24%	(2.12–2.31)
Circulatory and respiratory causes		10.65%	(9.93–11.33)	9.55%	(8.88–10.18)	1.10%	(1.05–1.15)
	Men	7.54%	(6.68–8.31)	6.72%	(5.94–7.43)	0.82%	(0.74–0.89)
	Women	13.50%	(12.44–14.42)	12.15%	(11.17–13.02)	1.34%	(1.27–1.40)
							
**B. Attributable number of deaths**							
		**Heat**		**Moderate heat**		**Extreme heat**	
		**AN**	**95% CI**	**AN**	**95% CI**	**AN**	**95% CI**
Circulatory causes		38,287	(35,192–41,174)	34,281	(31,453–36,935)	4,006	(3,738–4,239)
	Men	10,693	(8,826–12,428)	9,472	(7,784–11,064)	1,221	(1,042–1,363)
	Women	27,790	(25,498–29,995)	25,021	(22,921–27,075)	2,769	(2,577–2,920)
Respiratory causes		20,120	(18,288–21,664)	18,147	(16,410–19,623)	1,972	(1,878–2,041)
	Men	9,117	(8,117–10,046)	8,222	(7,302–9,093)	895	(814–953)
	Women	11,704	(10,011–12,818)	10,624	(8,987–11,703)	1,080	(1,024–1,115)
Circulatory and respiratory causes		57,814	(53,866–61,479)	51,844	(48,192–55,251)	5,971	(5,674–6,228)
	Men	19,377	(17,174–21,379)	17,273	(15,272–19,100)	2,104	(1,902–2,279)
	Women	38,543	(35,519–41,162)	34,705	(31,898–37,162)	3,838	(3,621–4,000)

Abbreviations: AF, attributable fraction; AN, attributable number; CI, confidence interval.

**Fig 5:**
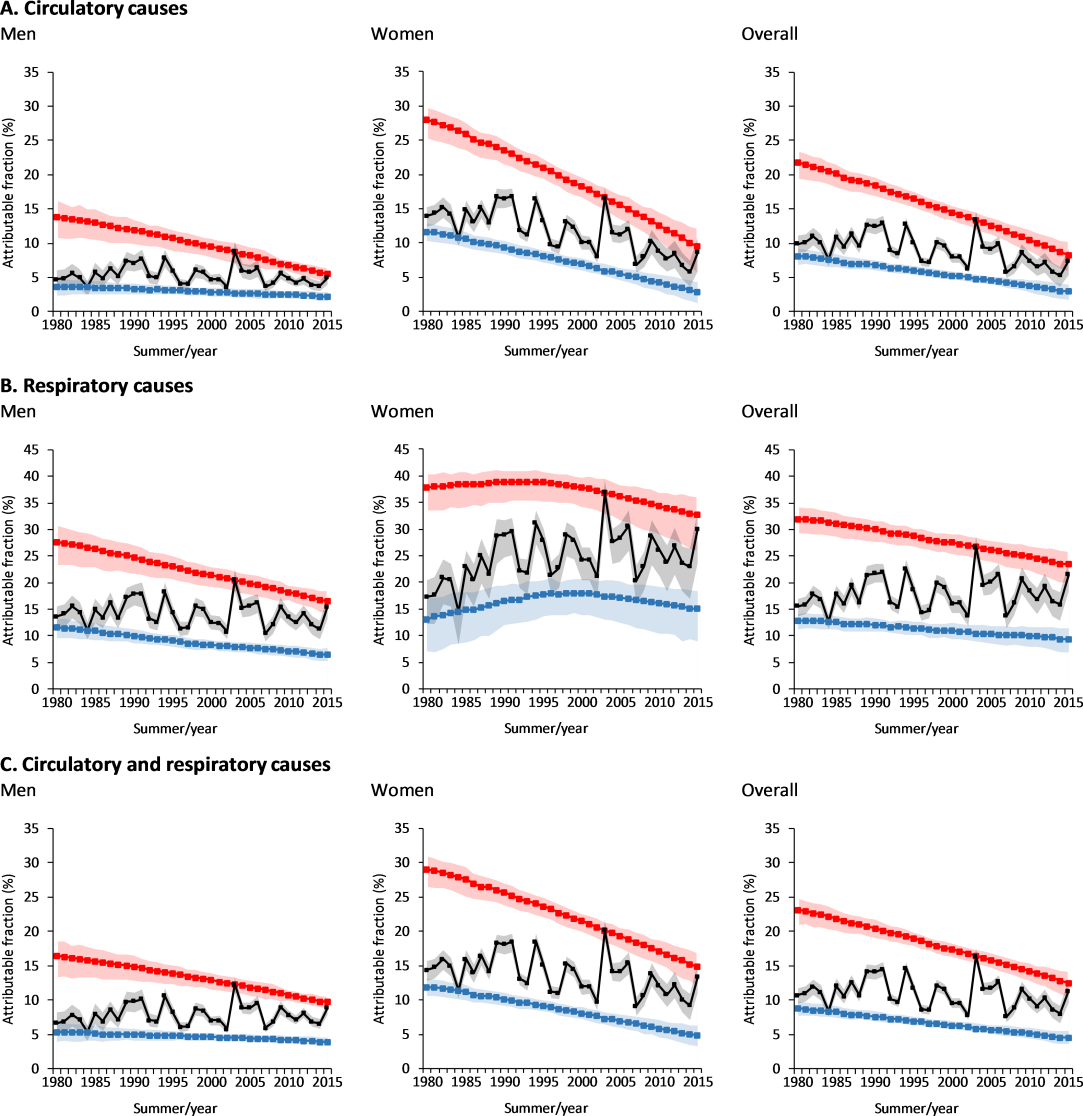
Temporal evolution of the mortality fraction attributable to heat (black). The blue and red lines result from applying the time-varying annual RR curves (1980–2015) to the daily time series of temperatures for summers 1984 and 2003, respectively. The shaded areas represent the 95% empirical confidence interval. RR, relative risk.

The black curves in Fig 5 depict the temporal evolution of the attributable fraction by cause of death and sex (see S8 Fig for the relative contribution of moderate and extreme heat). Regardless of the interannual variability and warming trend associated with the year-to-year changes in summer temperature, the heat-attributable fraction shows no clear and generalised upward trend during the study period. The only exception is found for respiratory diseases (for men and women together, and for women only), in which case the rather small reduction in RR only partially contributed to reducing the negative impact of increasing summer temperatures. Apart from these few exceptions, however, the general evolution shows that, regardless of the summer warming trend shown in Fig 1, the general lack of increasing trends in the attributable fraction is explained by the large decrease in the mortality RR shown in Figs 3 and 4.

The red and blue curves in Fig 5 show the temporal evolution of the attributable fraction to heat if the effect of long-term warming temperatures were removed. This hypothetical evolution is constructed by replacing the temperature time series by the daily time series of a given summer (i.e., 122 values corresponding to the four summer months here considered) that is repeated by construction for all the years in the study period. Thus, the blue and red lines result from predicting the attributable fraction by applying the time-varying annual RR curves (1980–2015) to the daily time series of temperatures for summers 1984 (i.e., the coldest during the study period) and 2003 (the warmest), respectively. In this way, given that more moderate summer temperatures were observed in 1984 and more extreme temperatures in 2003, the magnitude of the corresponding trends in the attributable fraction results to a large extent from the decreasing magnitude in the RR for the moderate or extreme temperature percentiles, respectively (cf. with Fig 4). In this way, the range between these lines as well as differences with regard to the central curves show the relative contribution of interannual temperature anomalies and year-to-year climate variability to the evolution of the attributable fraction; they also show a rough estimation of the potential impact range of near-future summer temperatures. For example, compared with the attributable fraction observed in 2003, the estimated impact of a 2003-like summer with the RR of 2015 would be 21.13% smaller for men, 28.16% for women, 38.31% for cardiovascular diseases, and 12.34% for respiratory diseases (i.e., decrease of the red curves between years 2003 and 2015).

## Discussion

To the best of our knowledge, this is the first study that comprehensively addresses the eventual impact of recent climate warming on summer mortality in Spain by cause of death and sex. The study pointed to a strong reduction in cause-specific and cause-sex mortality RR associated with summer temperatures for the last three and a half decades and, with the exception of respiratory diseases (for men and women together, and for women only), downward trends in heat-attributable deaths. These results strongly support the hypothesis that the observed warming trend in summer temperatures in Spain has not been paralleled by a general increase in the mortality fraction attributable to heat, as a result of substantial decline in population vulnerability to warm temperatures [11].

In this study, the effect of heat on mortality largely varied by cause of death. We observed a greater impact of heat on respiratory rather than circulatory mortality. This is in agreement with previous studies [24–27] and probably reflects the large health vulnerability among people with pre-existing or chronic respiratory diseases during hot periods [28]. It should be remembered that respiratory mortality accounted for only 21.1% of cardiorespiratory deaths during the study period, but the observed rise in the relative prevalence of respiratory diseases over recent decades might continue in the near future and keep increasing the relative percentage of the population susceptible to heat-related respiratory mortality. The underlying physiological mechanisms behind the effect of heat on mortality from circulatory and respiratory causes are not well known yet, but they seem to be largely mediated by a thermoregulatory pathway.

We assessed circulatory- and respiratory-specific mortality by sex, and we found that women were systematically more at risk of dying from heat. This finding has been reported in many previous articles analysing the effect through the whole range of hot temperatures [27,29–31] and in many studies based on extreme heat events [16,32–35]. These quantitative differences between men and women may partially arise from physiological characteristics in body temperature regulation between males and females [36]. However, most of these differences could simply be attributed to existing sociodemographic characteristics of the society (e.g., differences between men and women in age pyramid, life expectancy, or social isolation). Previous studies have actually shown that this type of difference can in some cases result in gender being an important variable to predict the mortality risk; i.e., 64% of the deaths were women during the 2003 heat wave in France [33]. The gender gap was, however, found to be reduced by the group of planned adaptation measures implemented just after this record-breaking episode [16]: while 60% of the deaths were expected to be women during the following major heat wave in 2006, gender differences in observed mortality were significantly lower; i.e., 53% of the deaths were women.

The heat attributable fractions reported in our study are higher than those presented, for example, in Gasparrini et al. [37] and somewhat different, for example, from those reported by Carmona et al. [38]. However, these results are not comparable, because in our study, mortality data are analysed only for circulatory and respiratory causes, which are two of the main groups associated with ambient temperatures (see, for example, Basagaña et al. [39]). Instead, in Gasparrini et al. [37], mortality data for Spain are analysed for all causes of death together, and in Carmona et al. [38] for natural causes, including many causes of death that are not largely associated with ambient temperatures. As a result, the attributable fraction (i.e., the ratio between attributable deaths and total deaths) is understandably higher in our study than in Gasparrini et al. [37] and Carmona et al. [38]. We note that other methodological differences might explain (to a lower extent) these differences; e.g., the time period of data is different (taking into account that the relative risk decreases with time).

The temporal evolution of heat-related mortality risks here found is, in general, consistent with those reported by previous studies in some other countries [12–15], which provide evidence for a decrease in vulnerability to climate warming despite the ageing of societies. For example, in Spain, the proportion of people aged over 64 years increased from 11.6% to 15.0% in men and from 15.9% to 19.6% in women between 1991 and 2011 [40]. The general downward trend in mortality risks has been attributed by some investigators to socioeconomic development and structural transformations, such as improvements in housing and healthcare services [12–15], or even to specific public health interventions [16–18]. The large socioeconomic advances that occurred in Spain during the last decades might have also contributed to this response, thus reducing the effect of mortality risks over time. For example, the gross domestic product (from €8,798 per capita in 1991 to €22,813 in 2009), the life expectancy at birth (from 77.08 years to 81.58), the expenditure in healthcare (from €605 per capita to €2,182) and social protection (from €1,845 per capita to €5,746), and the number of doctors (from 3,930 per million inhabitants to 4,760 per million inhabitants) have all largely increased in Spain [41]. In addition, the use of air conditioning, which has been postulated as a major contributor to the reduction in heat-related mortality in the United States [13], has also experienced a strong increase in Spanish households within the analysed period (from 5.3% to 35.5%) [42].

Another potential contributing factor to the reduction of the mortality risks might have been the ‘National plan for preventive actions against the effects of excess temperatures on health’ from the Spanish Ministry of Health [43], which was implemented in 2004, just after the 2003 summer heat wave, in order to minimise the negative effects of summer extreme temperatures on the population’s health, particularly among vulnerable groups such as the elderly, children, people with chronic disease, and disadvantaged persons. Nonetheless, we do not see a change in the slope of the generally linear declining trend of RR and attributable fraction in the mid-2000s, which seems to indicate that this measure had at most a minor beneficial impact (specific analyses will be performed elsewhere). The cause-specific and cause-sex attributable numbers all showed a rather linear downward trend during the study period as a result of the strong decrease in RR, which was largest for the warmest temperatures. Although the range corresponding to moderate hot temperatures had a comparatively lower RR, it included the majority of days in the series, accounting for most of the overall deaths caused by heat. In that regard, public health interventions should also be directed towards non-extreme temperatures, to include the whole range of moderate conditions above the MMT [37].

Finally, some limitations of the study deserve to be mentioned. First, we were not able to control for air pollution because of data unavailability. However, previous studies showed that effects of hot temperatures on mortality in the US were only slightly reduced after adjusting for air pollution [24]. Secondly, the study did not take into account the long-term changes in the age structure of the population, which will be analysed when a more complete dataset by age is available.

## Supporting information

S1 STROBE Checklist STROBE checklist. (PDF)

S1 Fig Sensitivity analysis for modelling choices. (PDF)

S2 Fig Temporal evolution of summer (June–September) deaths for 1980–2015. (PDF)

S3 Fig Temperature-mortality relationships for the 47 provincial capital cities in Spain. (PDF)

S4 Fig Estimated minimum mortality temperature for the 47 provincial capital cities in Spain. (PDF)

S5 Fig Relationship for the ensemble of the cities between MMT and summer mean temperature. MMT, minimum mortality temperature.
(PDF)

S6 Fig Temperature-mortality relationships predicted for 1980 (green) and 2015 (blue) in the 47 provincial capital cities. (PDF)

S7 Fig Temporal evolution of mortality RR at the 90th temperature percentile from the model with interaction (time-varying DLNM). DLNM, distributed lag nonlinear model; RR, relative risk.
(PDF)

S8 Fig Temporal evolution of mortality attributable to moderate and extreme heat for 1980–2015. (PDF)

S1 Analysis Plan Analysis plan. (PDF)

S1 Table Results of the multivariate Wald test for mortality RR curves. RR, relative risk.
(PDF)

## Abbreviations

BLUP: best linear unbiased prediction
CI: confidence interval
DLNM: distributed lag nonlinear model
ECA&D: European Climate Assessment and Dataset
INE: Spanish National Statistics Institute
MMT: minimum mortality temperature
RR: relative risk
STROBE: Strengthening the Reporting of Observational Studies in Epidemiology
