# Correction: Heat-related mortality trends under recent climate warming in Spain: A 36-year observational study

**DOI:** 10.1371/journal.pmed.1003627

**Published:** 2021-04-30

**Authors:** Hicham Achebak, Daniel Devolder, Joan Ballester

The following information is missing from the Funding statement: DD acknowledges financial support from the Spanish Ministry of Economy, Industry and Competitiveness through the R&D&i State Program; Ref. CSO2017-89721-R).
