# Supplementary material for: Heat-related mortality trends under recent climate warming in Spain: A 36-year observational study
Source: PLoS Med. 2018 Jul 24;15(7):e1002617. doi: 10.1371/journal.pmed.1002617 (PMC6057624; doi:10.1371/journal.pmed.1002617)

S1 Fig. Sensitivity analysis for modelling choices

A. Knots for exposure-response

Men Women Overall

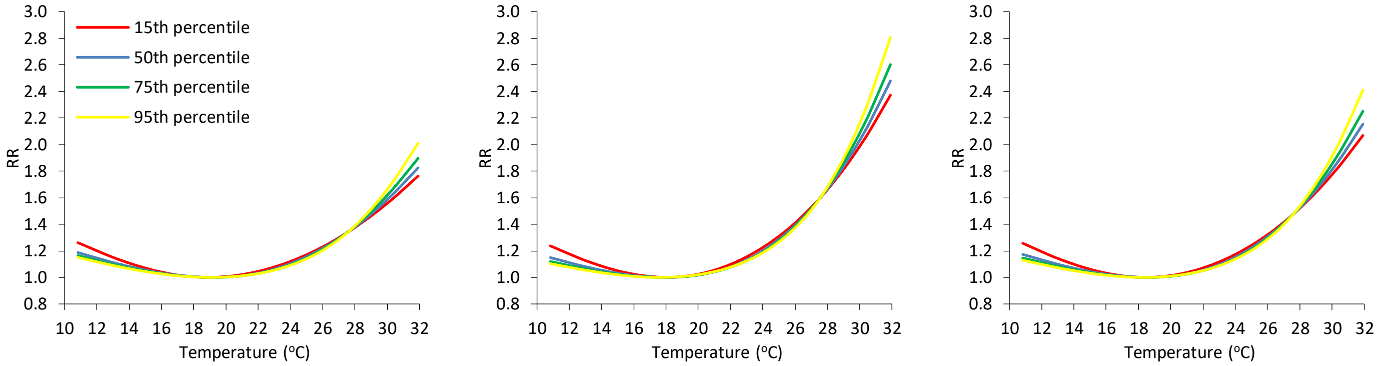

B. Lag-period

Men Women Overall

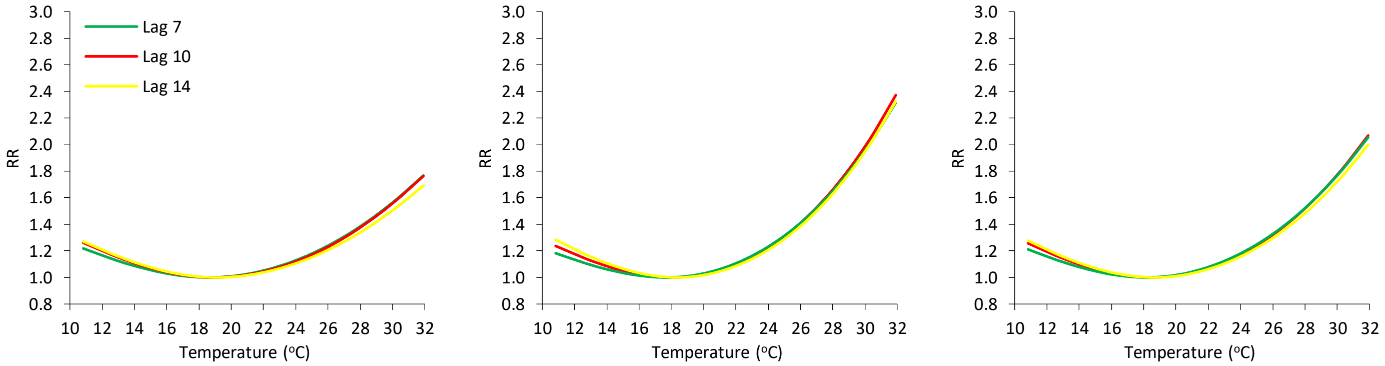

C. Degrees of freedom for seasonal trend

Men Women Overall

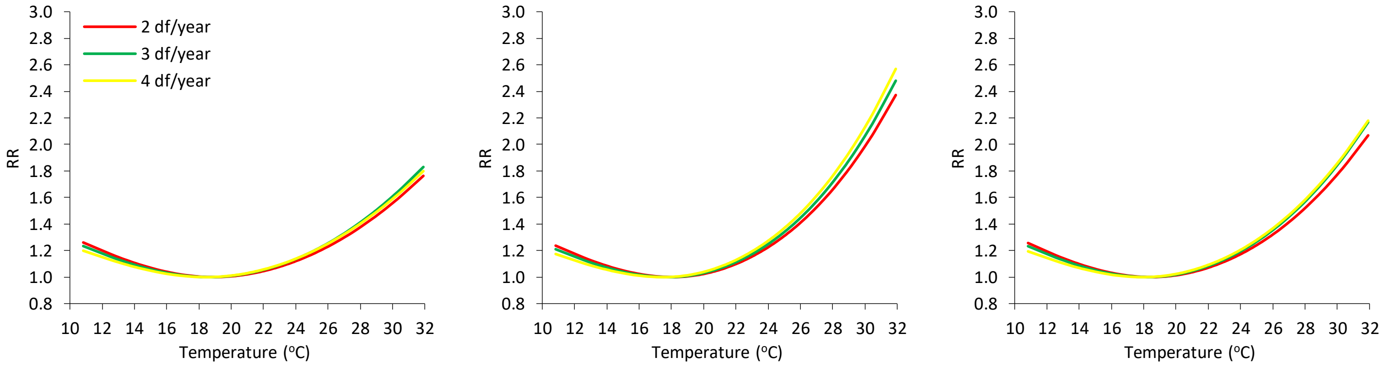

D. Degrees of freedom for long-term trend

Men Women Overall

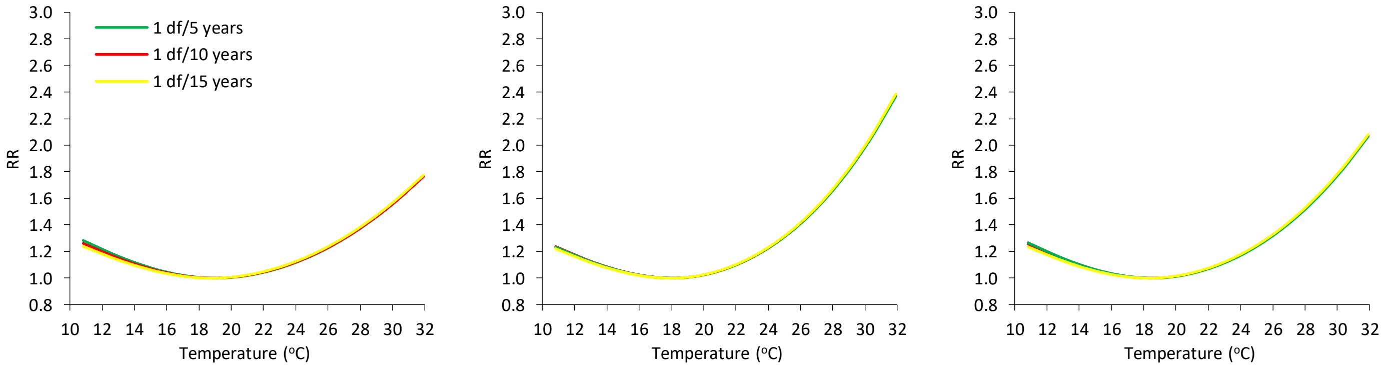

Supplement: S1 Fig — (PDF) [file pmed.1002617.s002.pdf]
