# Supplementary material for: Heat-related mortality trends under recent climate warming in Spain: A 36-year observational study
Source: PLoS Med. 2018 Jul 24;15(7):e1002617. doi: 10.1371/journal.pmed.1002617 (PMC6057624; doi:10.1371/journal.pmed.1002617)

S2 Fig. Temporal evolution of summer (June-September) deaths for 1980-2015

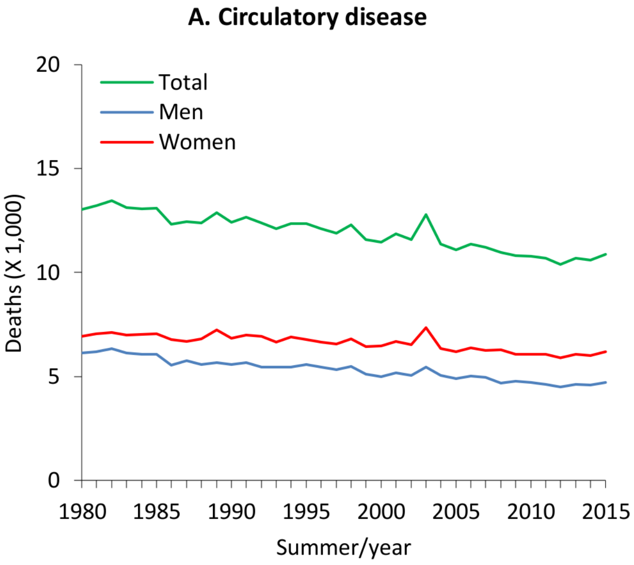

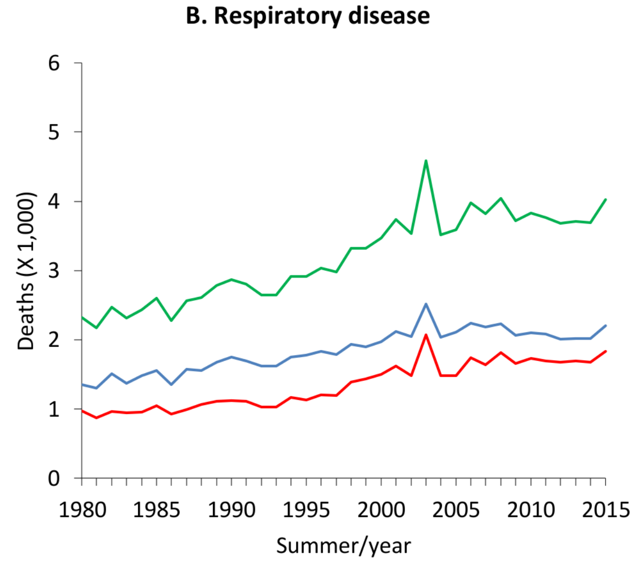

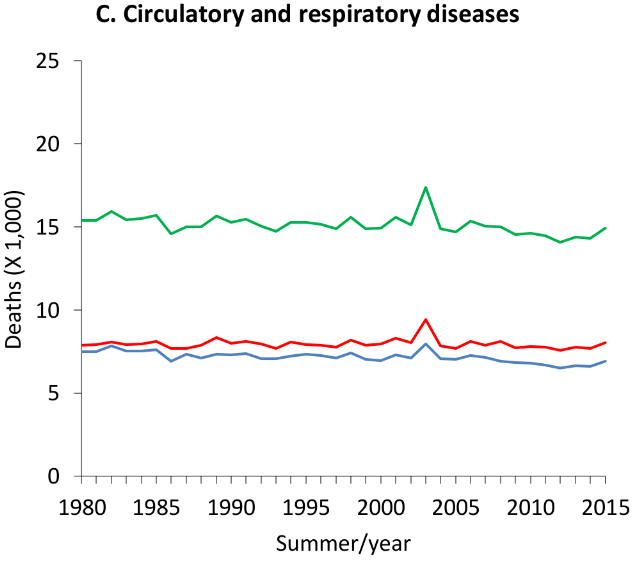

Supplement: S2 Fig — (PDF) [file pmed.1002617.s003.pdf]
