# Supplementary material for: Heat-related mortality trends under recent climate warming in Spain: A 36-year observational study
Source: PLoS Med. 2018 Jul 24;15(7):e1002617. doi: 10.1371/journal.pmed.1002617 (PMC6057624; doi:10.1371/journal.pmed.1002617)

S3 Fig. Temperature-mortality relationships for the 47 provincial capital cities in Spain

Circulatory and respiratory diseases

Overall

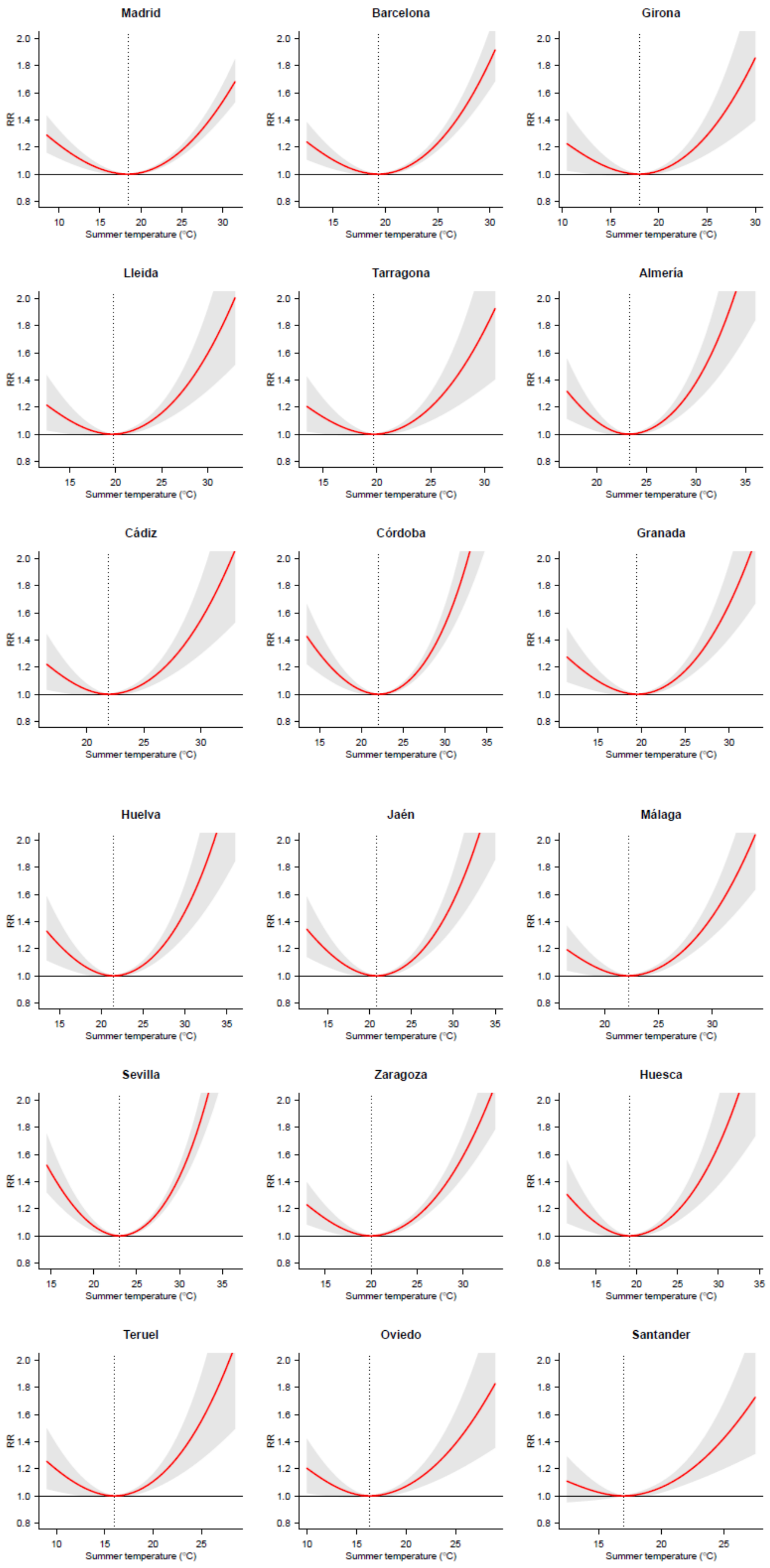

S3 Fig. Temperature-mortality relationships for the 47 provincial capital cities in Spain

Circulatory and respiratory diseases

Overall

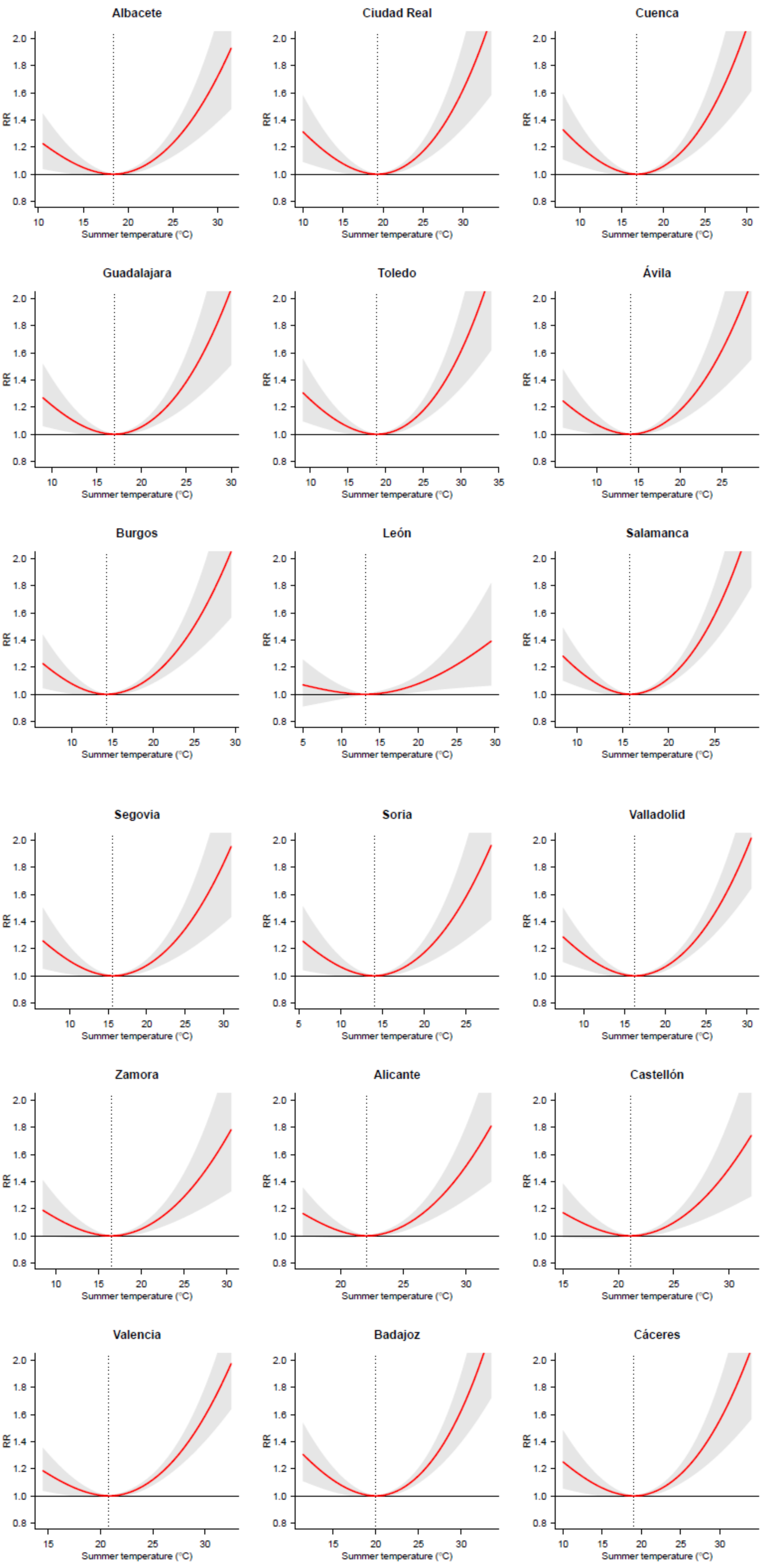

S3 Fig. Temperature-mortality relationships for the 47 provincial capital cities in Spain

Circulatory and respiratory diseases

Overall

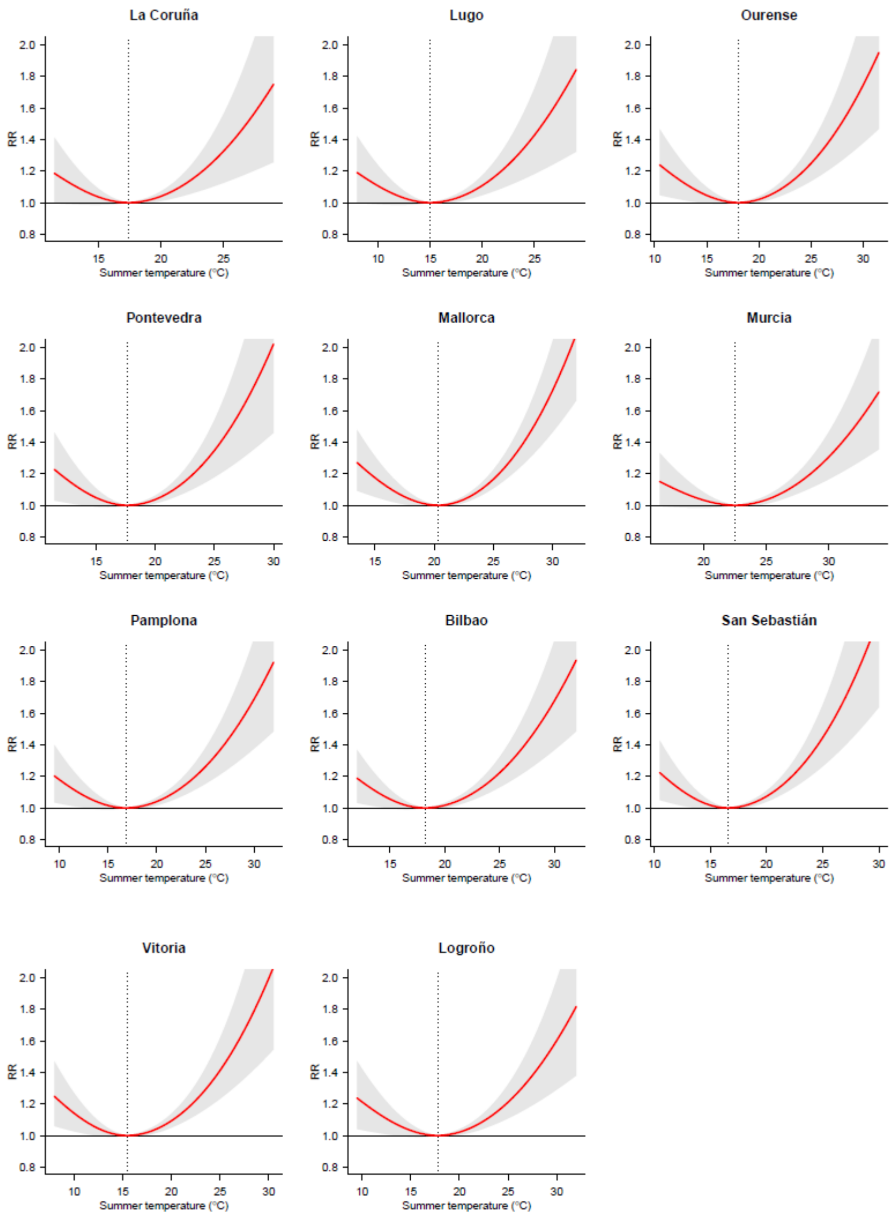

S3 Fig. Temperature-mortality relationships for the 47 provincial capital cities in Spain

Circulatory and respiratory diseases

Men

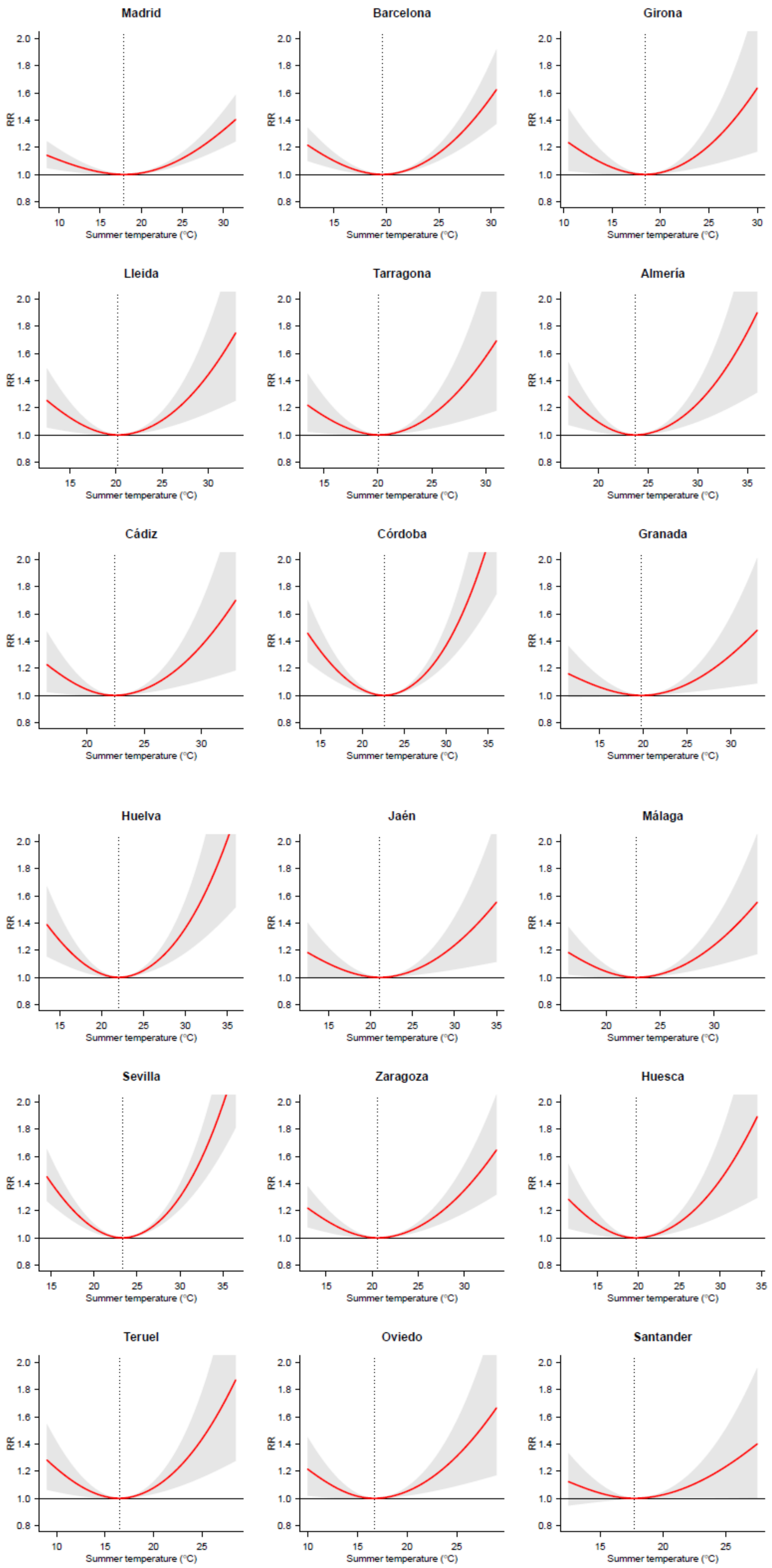

S3 Fig. Temperature-mortality relationships for the 47 provincial capital cities in Spain

Circulatory and respiratory diseases

Men

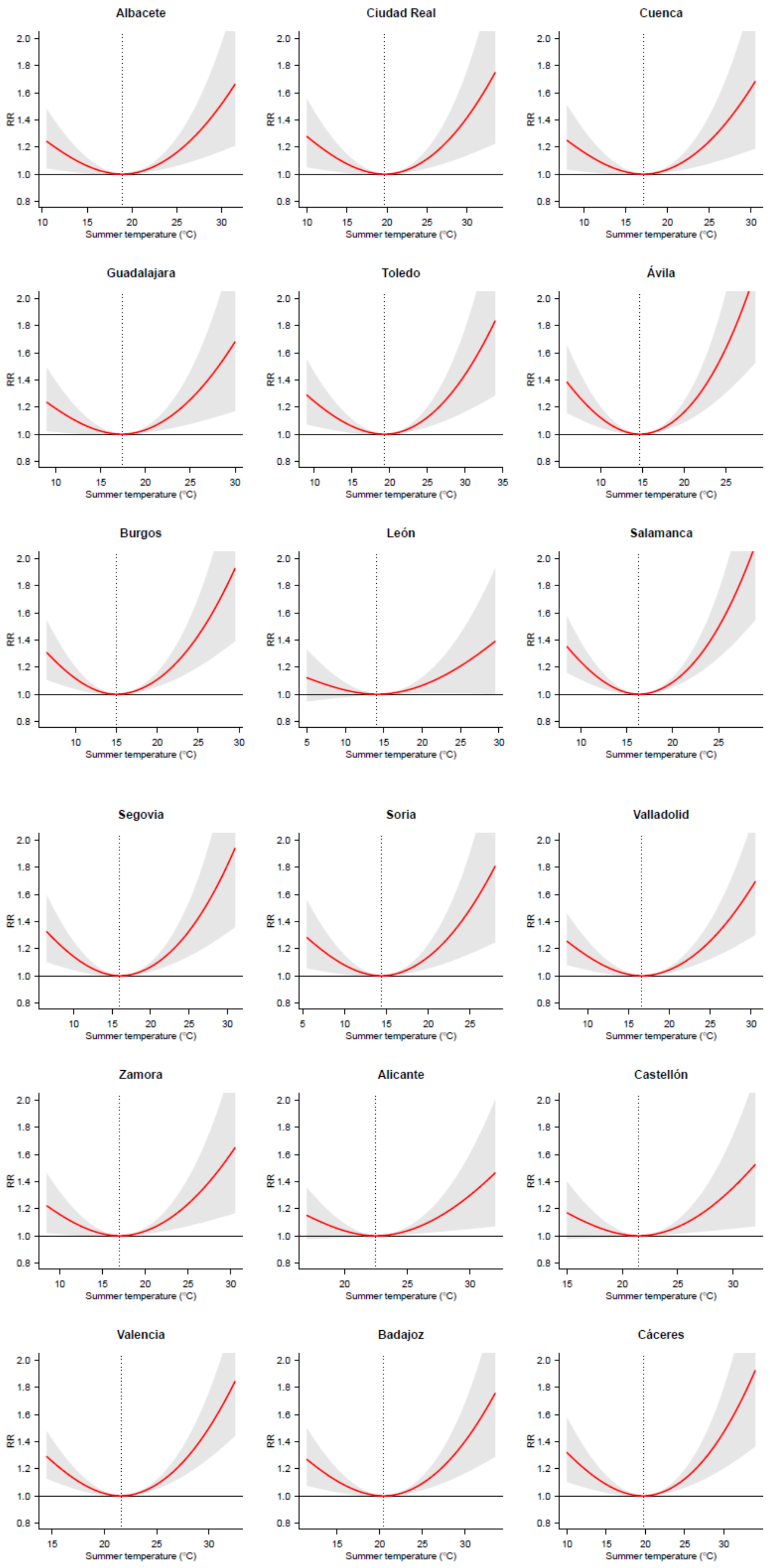

S3 Fig. Temperature-mortality relationships for the 47 provincial capital cities in Spain

Circulatory and respiratory diseases

Men

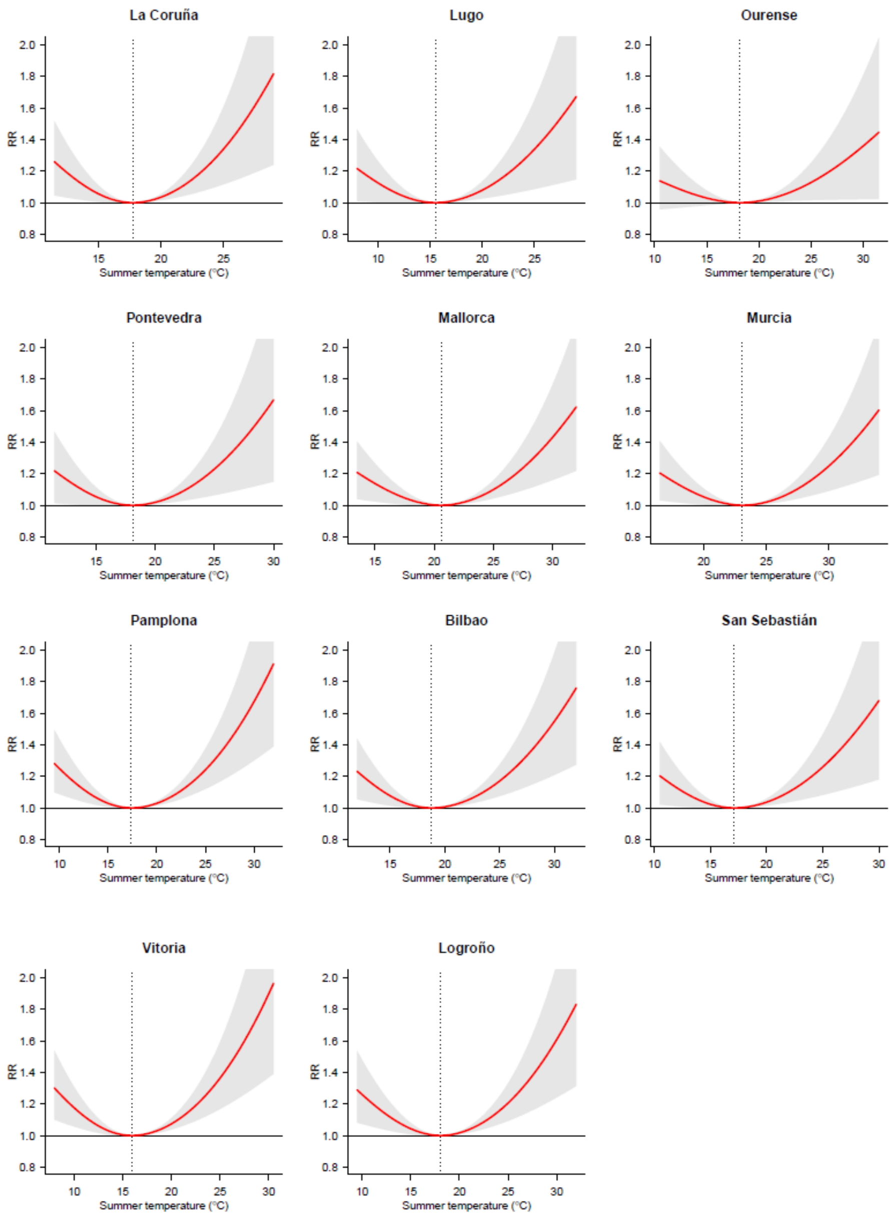

S3 Fig. Temperature-mortality relationships for the 47 provincial capital cities in Spain

Circulatory and respiratory diseases

Women

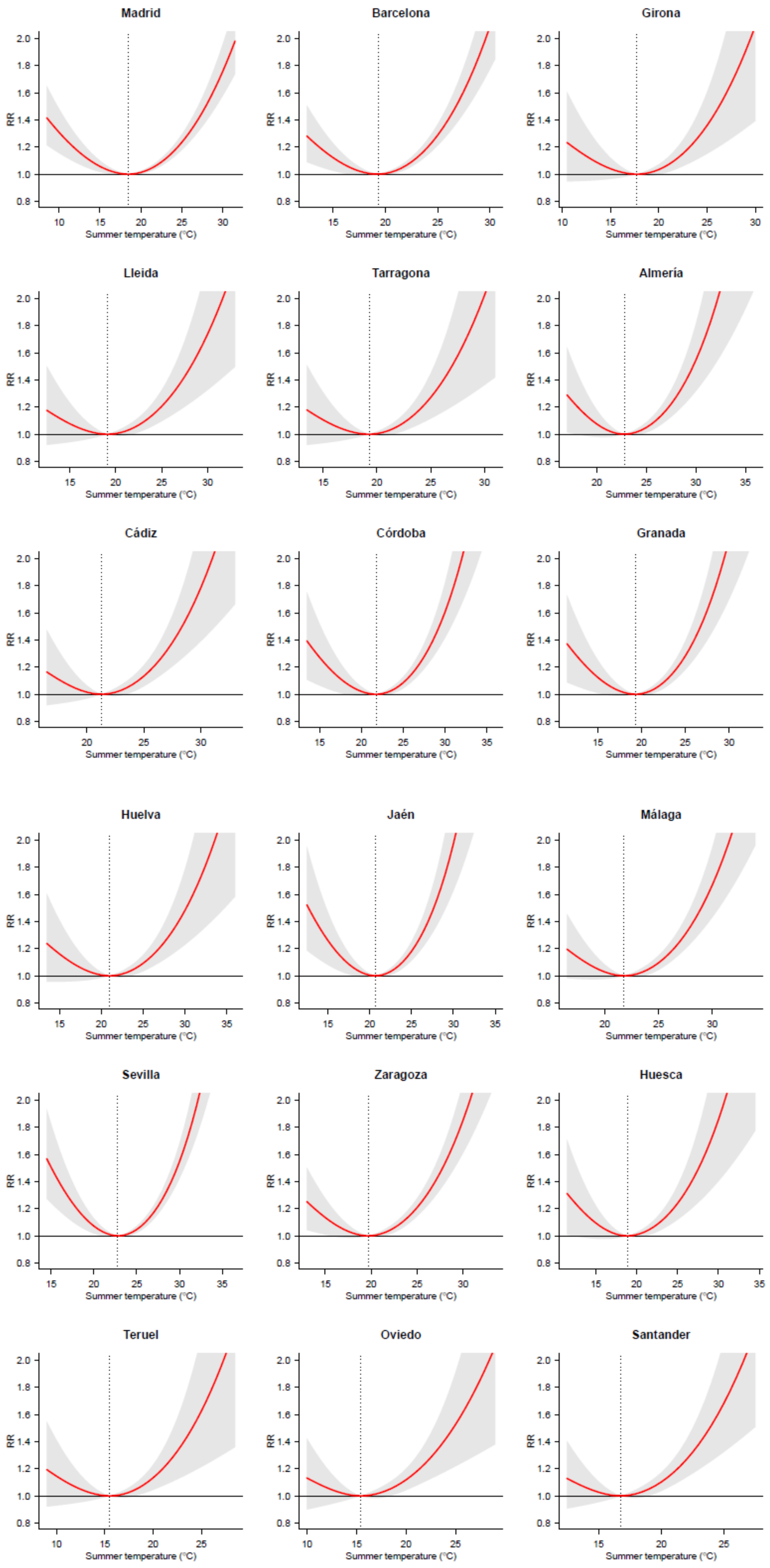

S3 Fig. Temperature-mortality relationships for the 47 provincial capital cities in Spain

Circulatory and respiratory diseases

Women

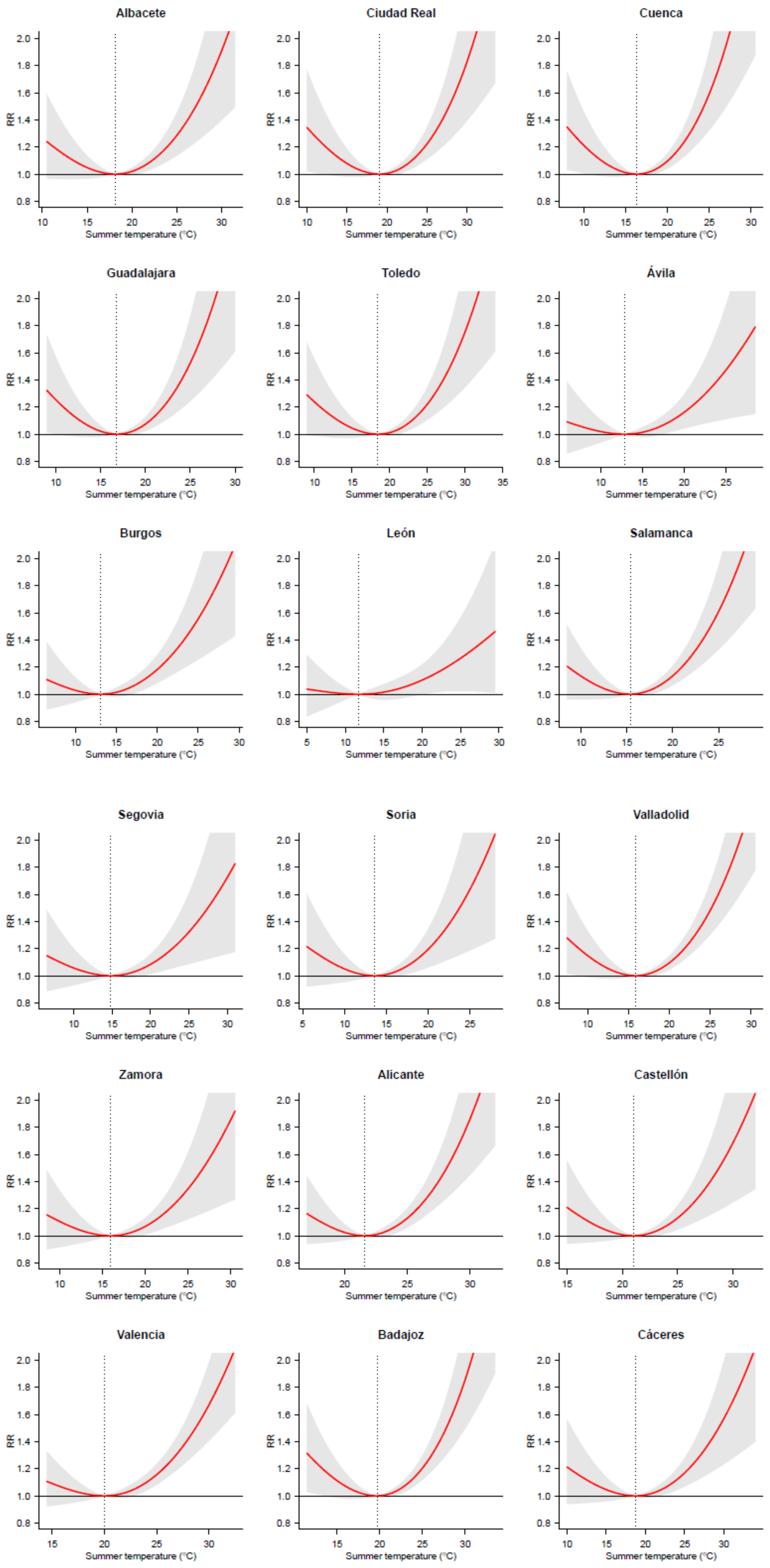

S3 Fig. Temperature-mortality relationships for the 47 provincial capital cities in Spain

Circulatory and respiratory diseases

Women

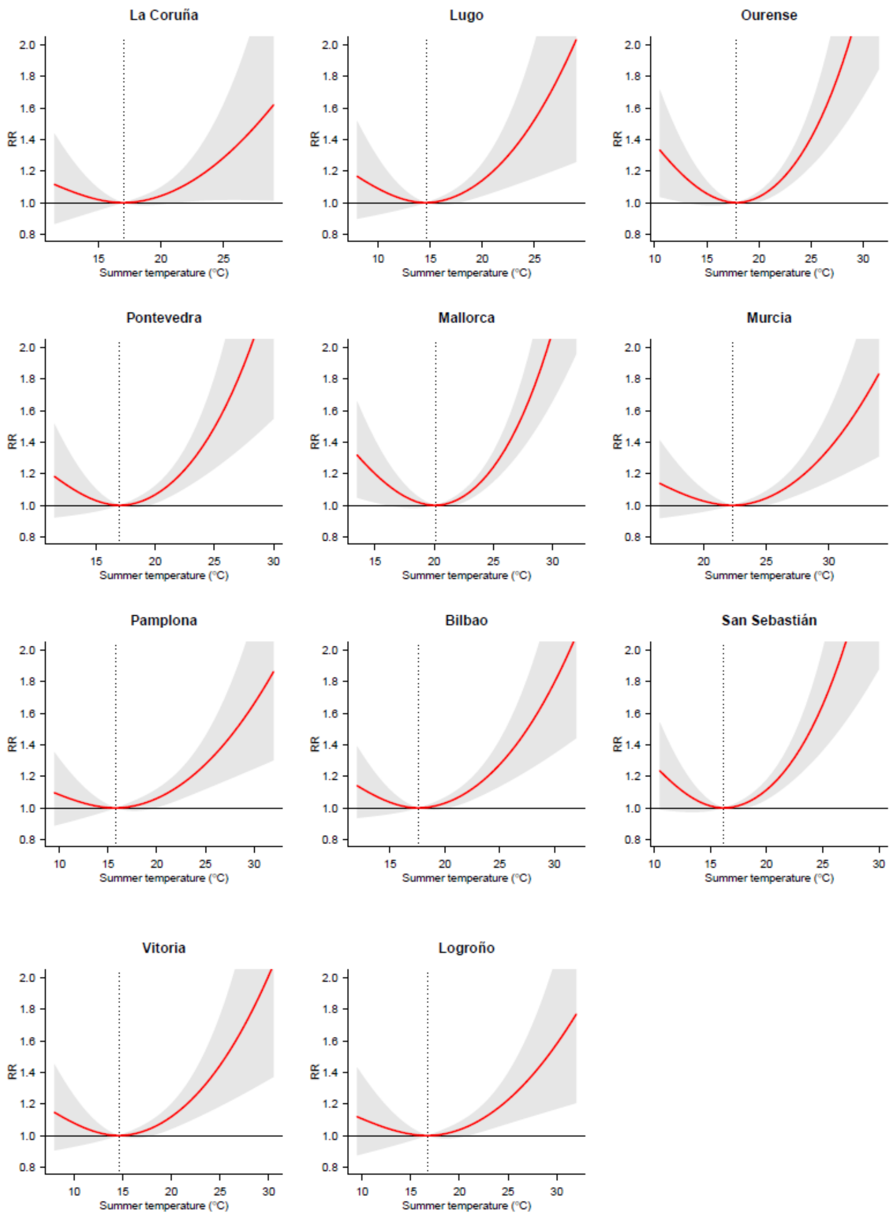

S3 Fig. Temperature-mortality relationships for the 47 provincial capital cities in Spain

Circulatory diseases

Overall

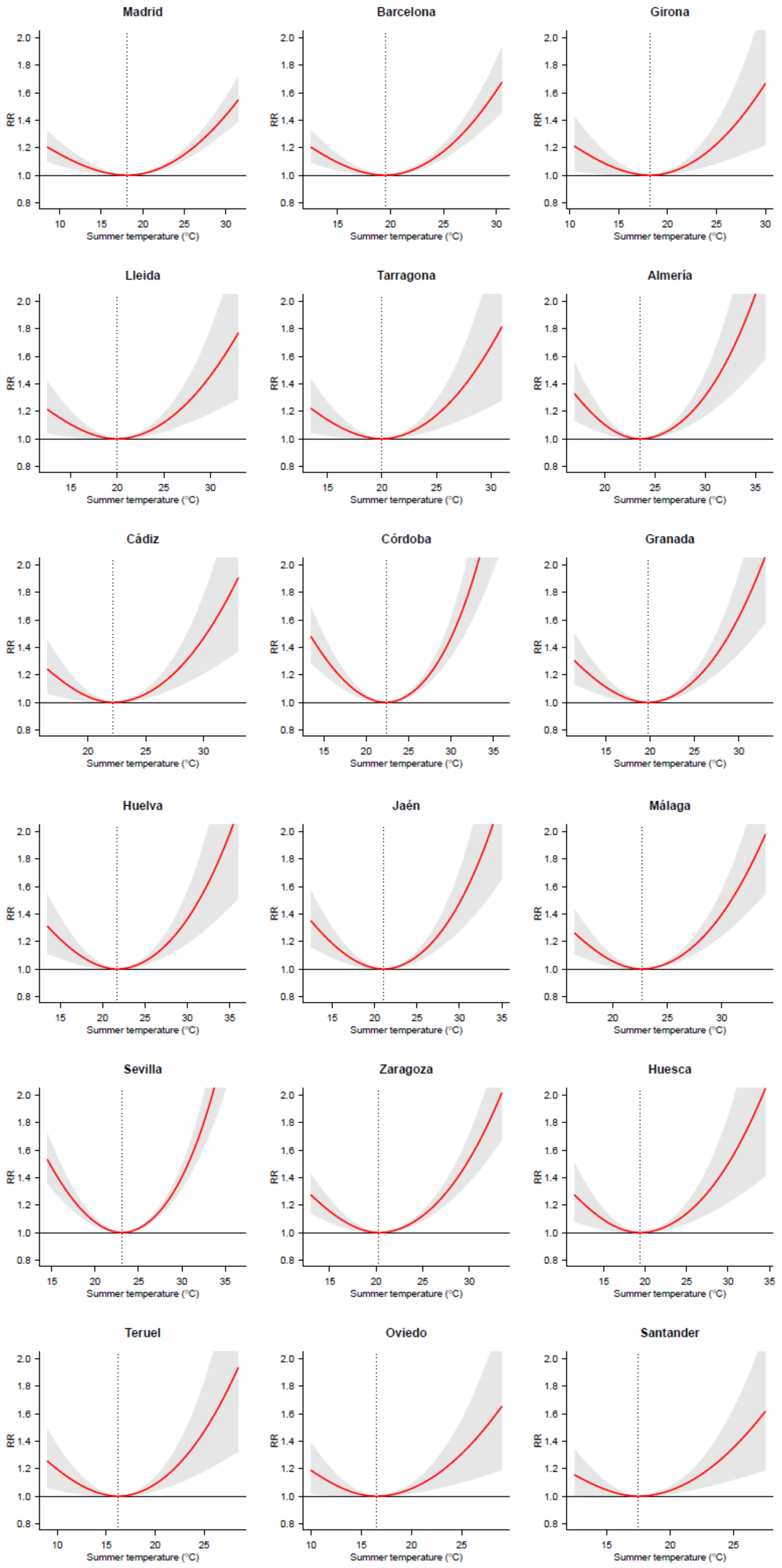

S3 Fig. Temperature-mortality relationships for the 47 provincial capital cities in Spain

Circulatory diseases

Overall

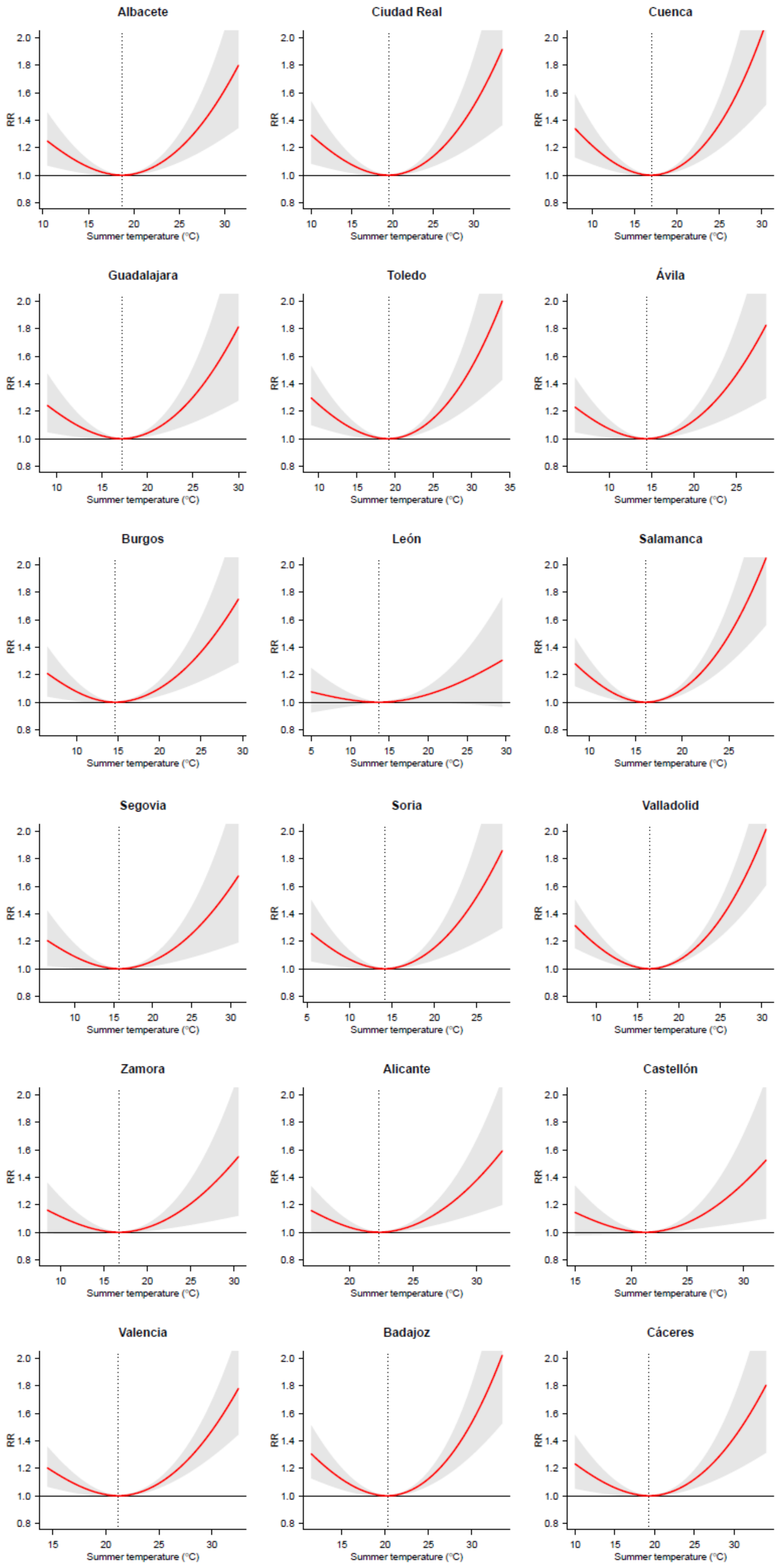

S3 Fig. Temperature-mortality relationships for the 47 provincial capital cities in Spain

Circulatory diseases

Overall

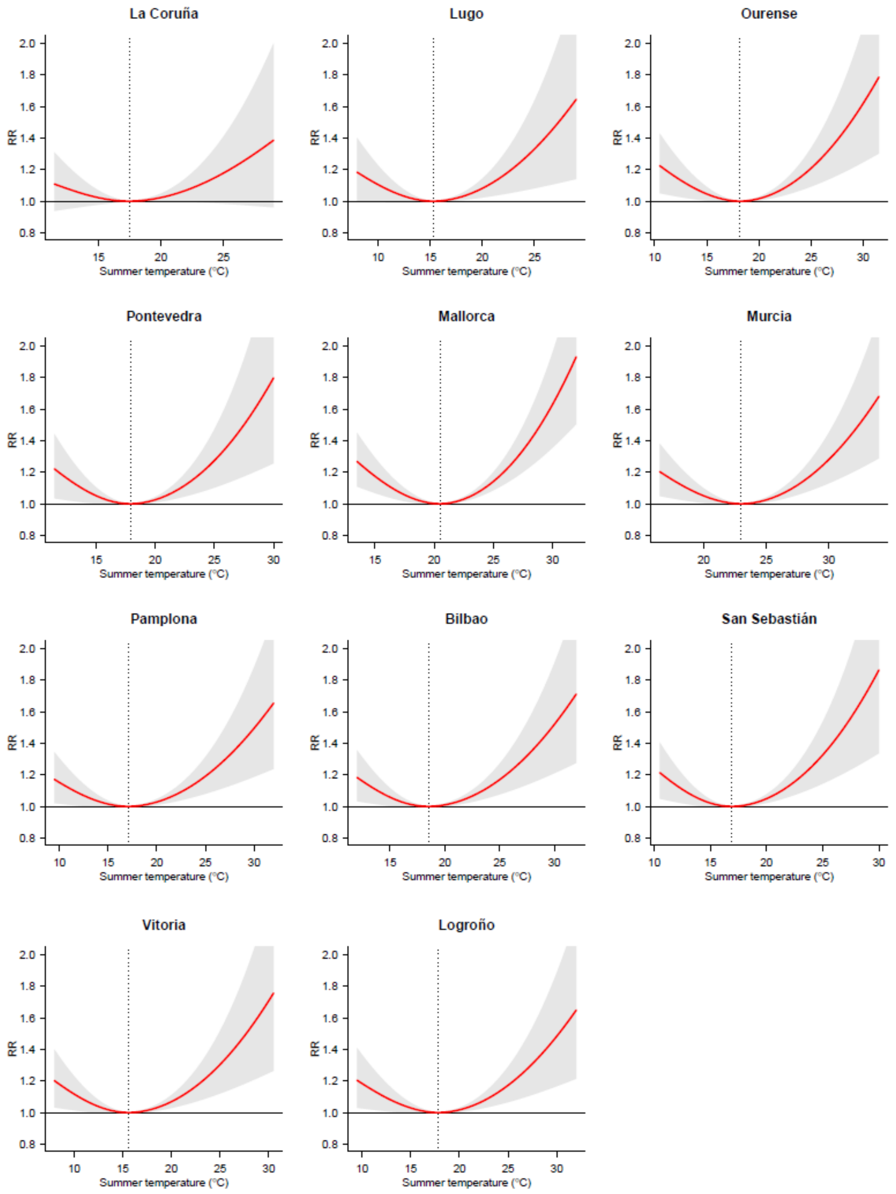

S3 Fig. Temperature-mortality relationships for the 47 provincial capital cities in Spain

Circulatory diseases

Men

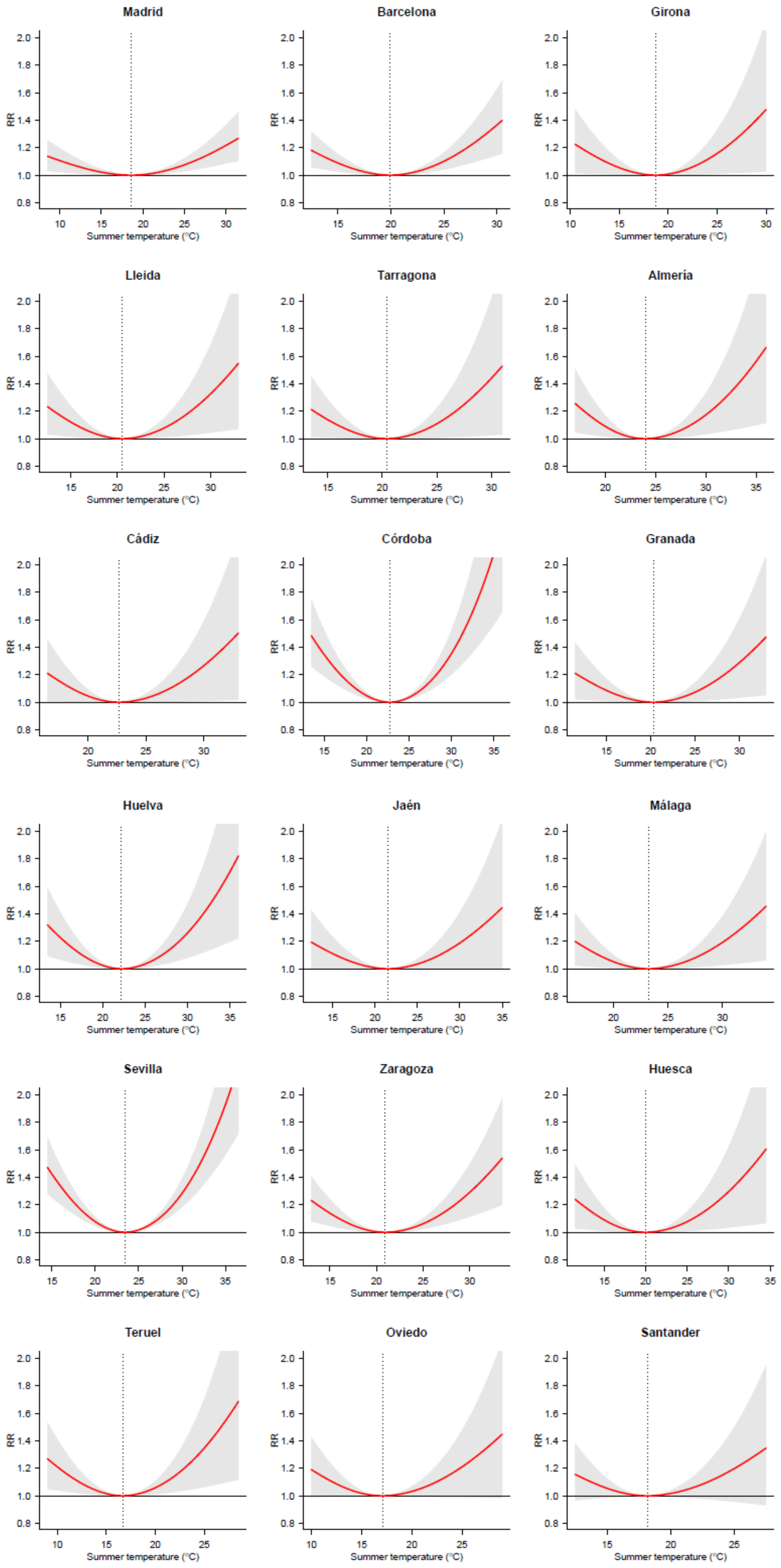

S3 Fig. Temperature-mortality relationships for the 47 provincial capital cities in Spain

Circulatory diseases

Men

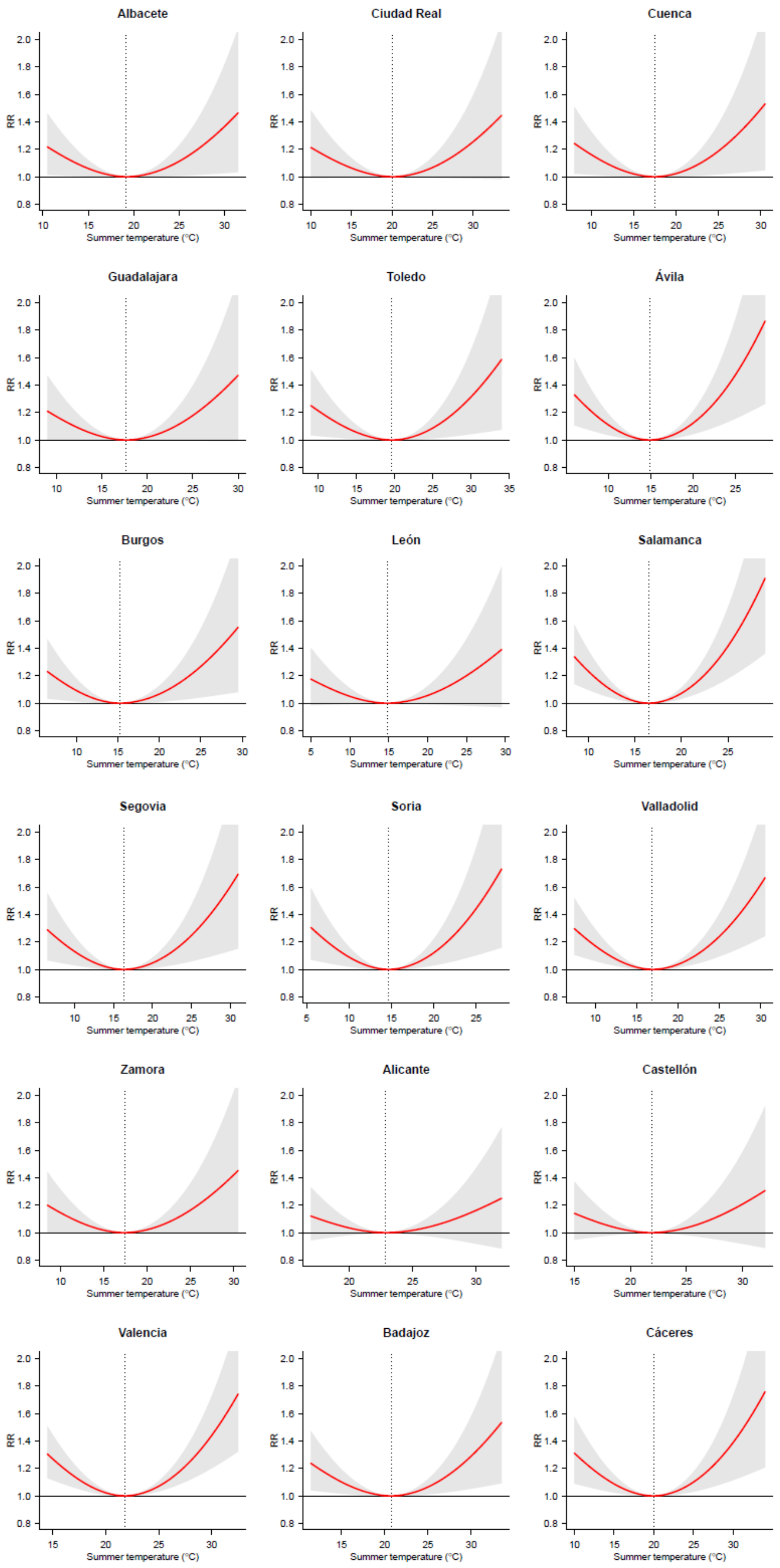

S3 Fig. Temperature-mortality relationships for the 47 provincial capital cities in Spain

Circulatory diseases

Men

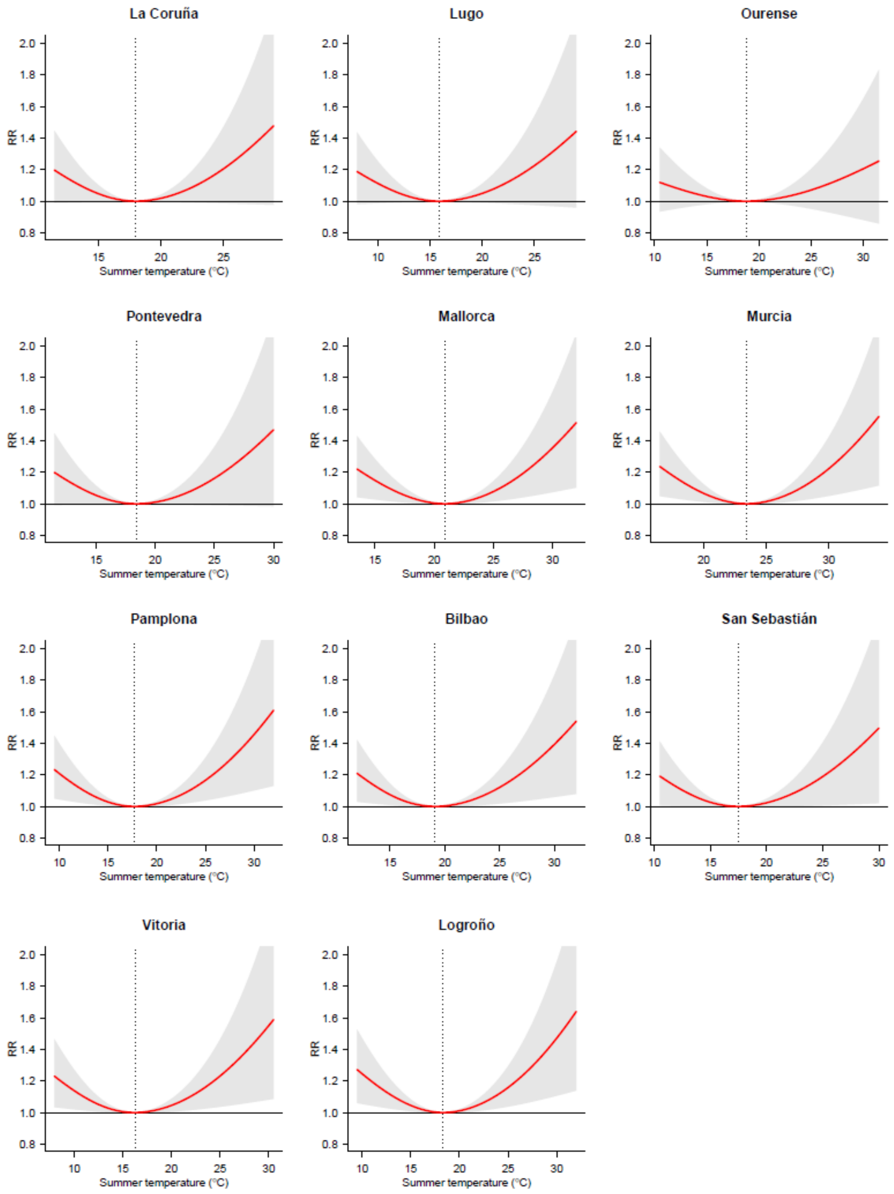

S3 Fig. Temperature-mortality relationships for the 47 provincial capital cities in Spain

Circulatory diseases

Women

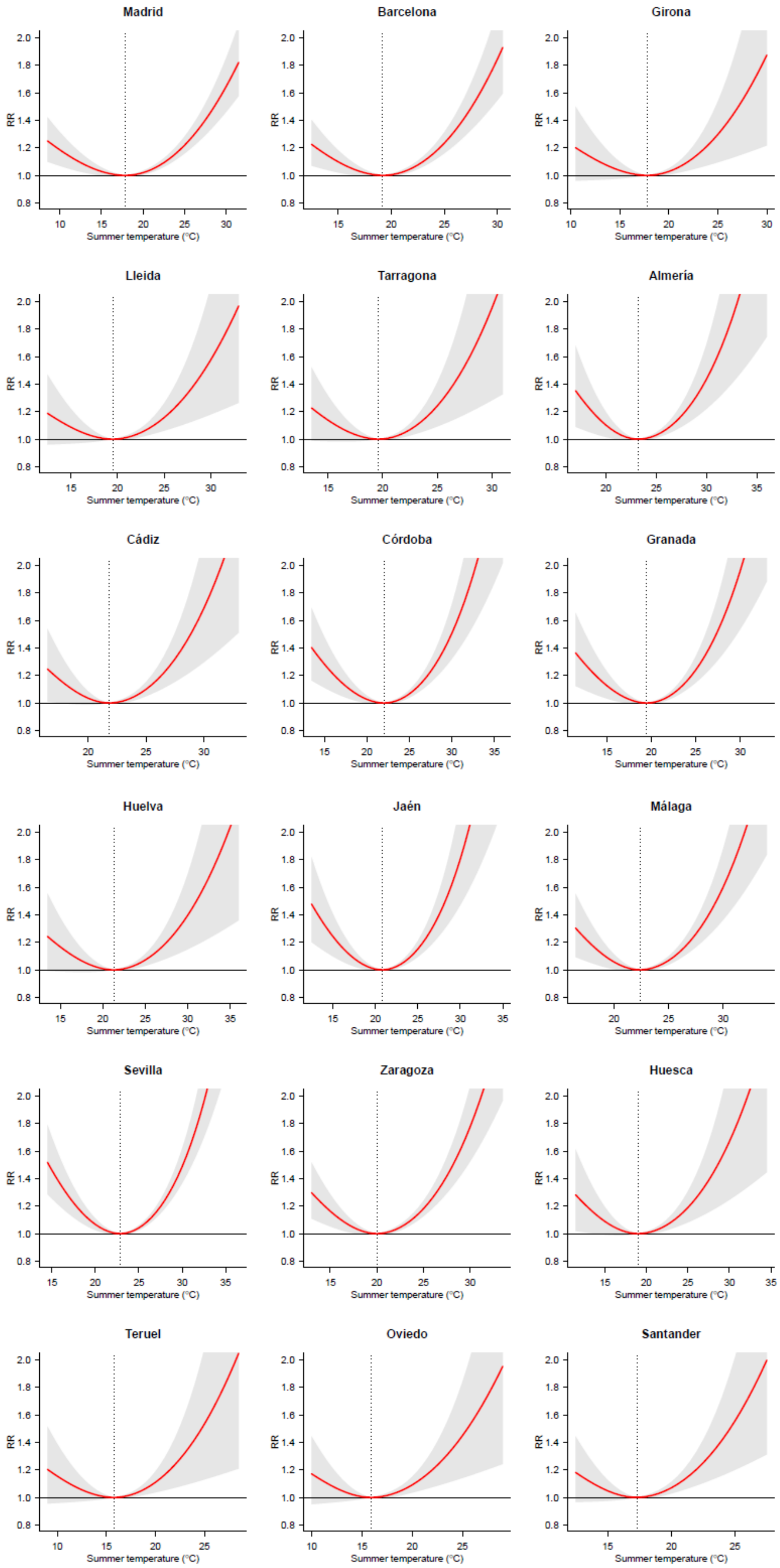

S3 Fig. Temperature-mortality relationships for the 47 provincial capital cities in Spain

Circulatory diseases

Women

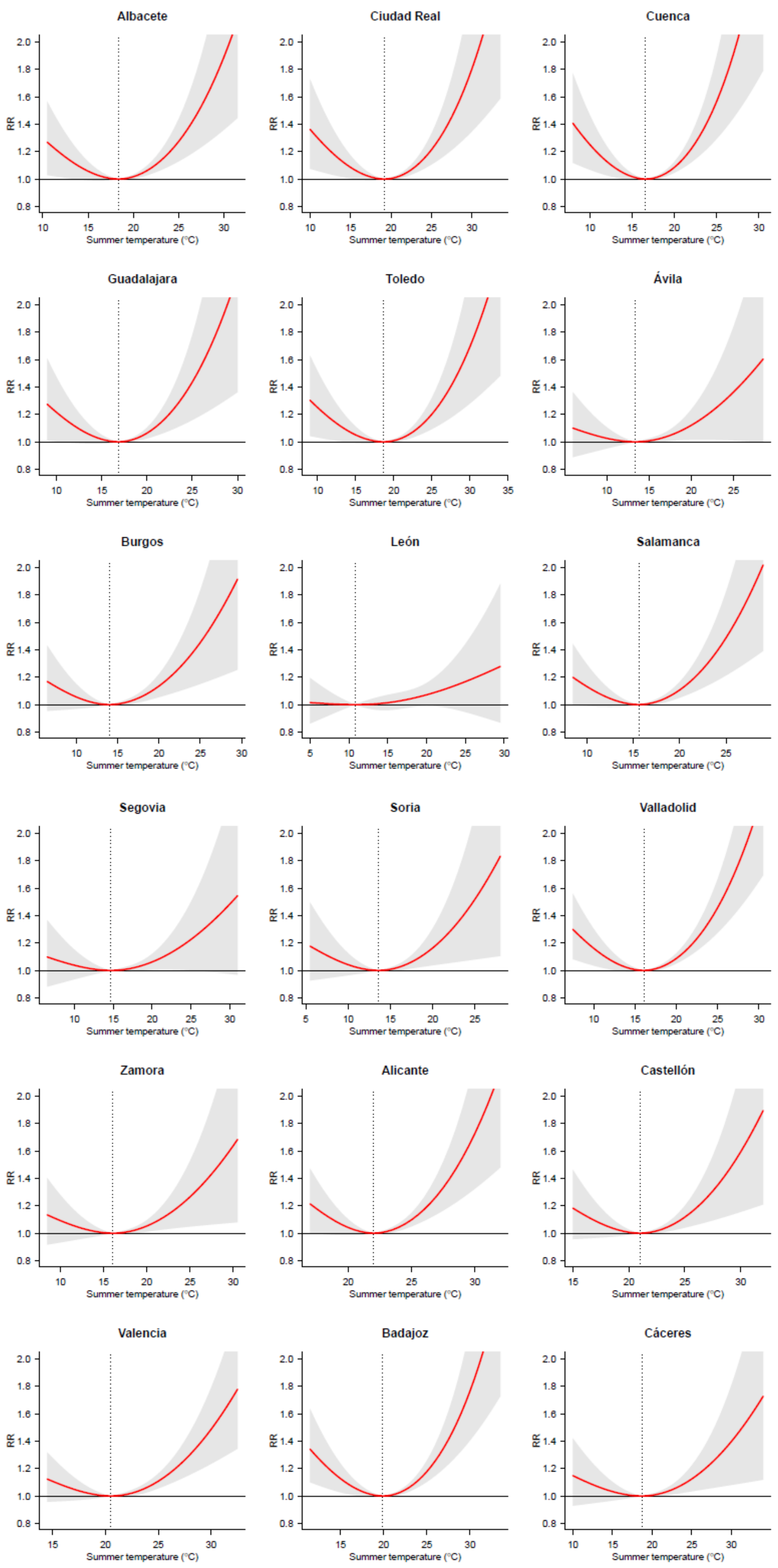

S3 Fig. Temperature-mortality relationships for the 47 provincial capital cities in Spain

Circulatory diseases

Women

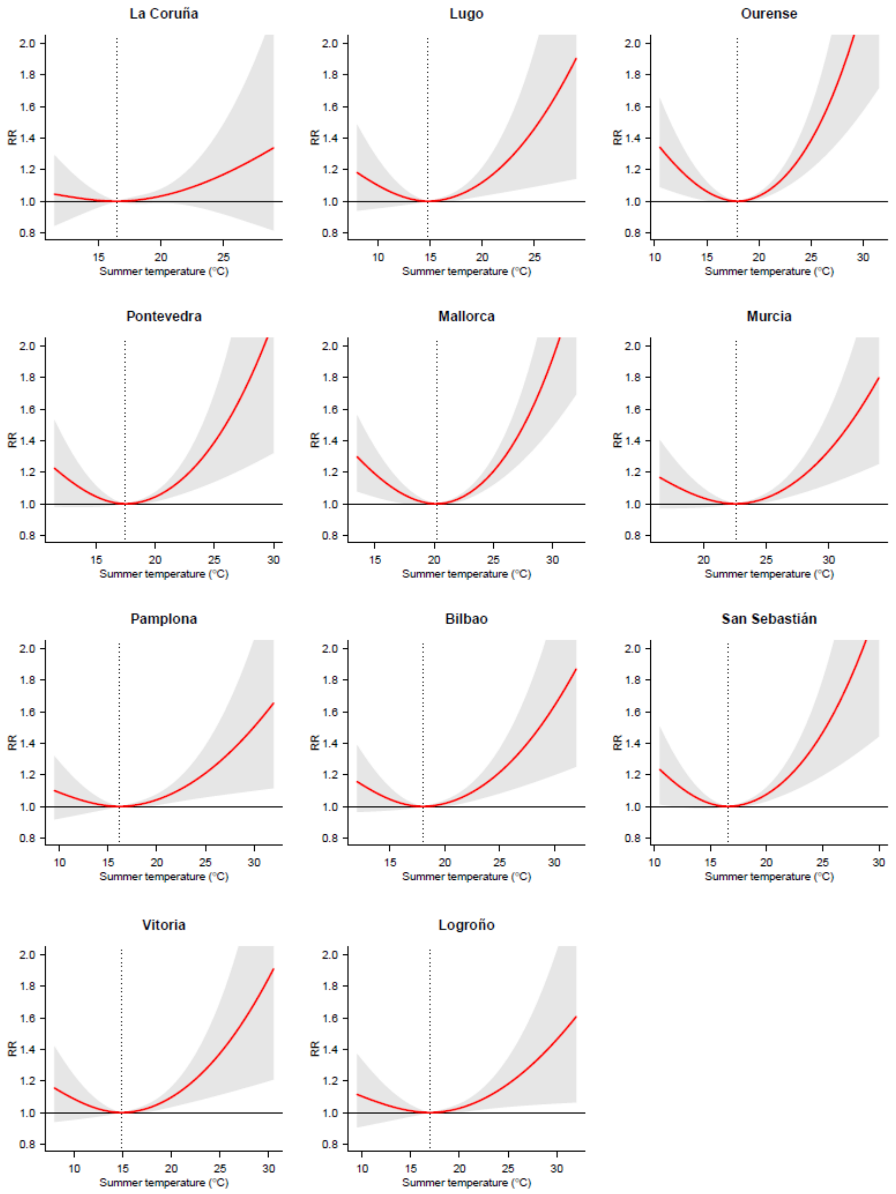

S3 Fig. Temperature-mortality relationships for the 47 provincial capital cities in Spain

Respiratory diseases

Overall

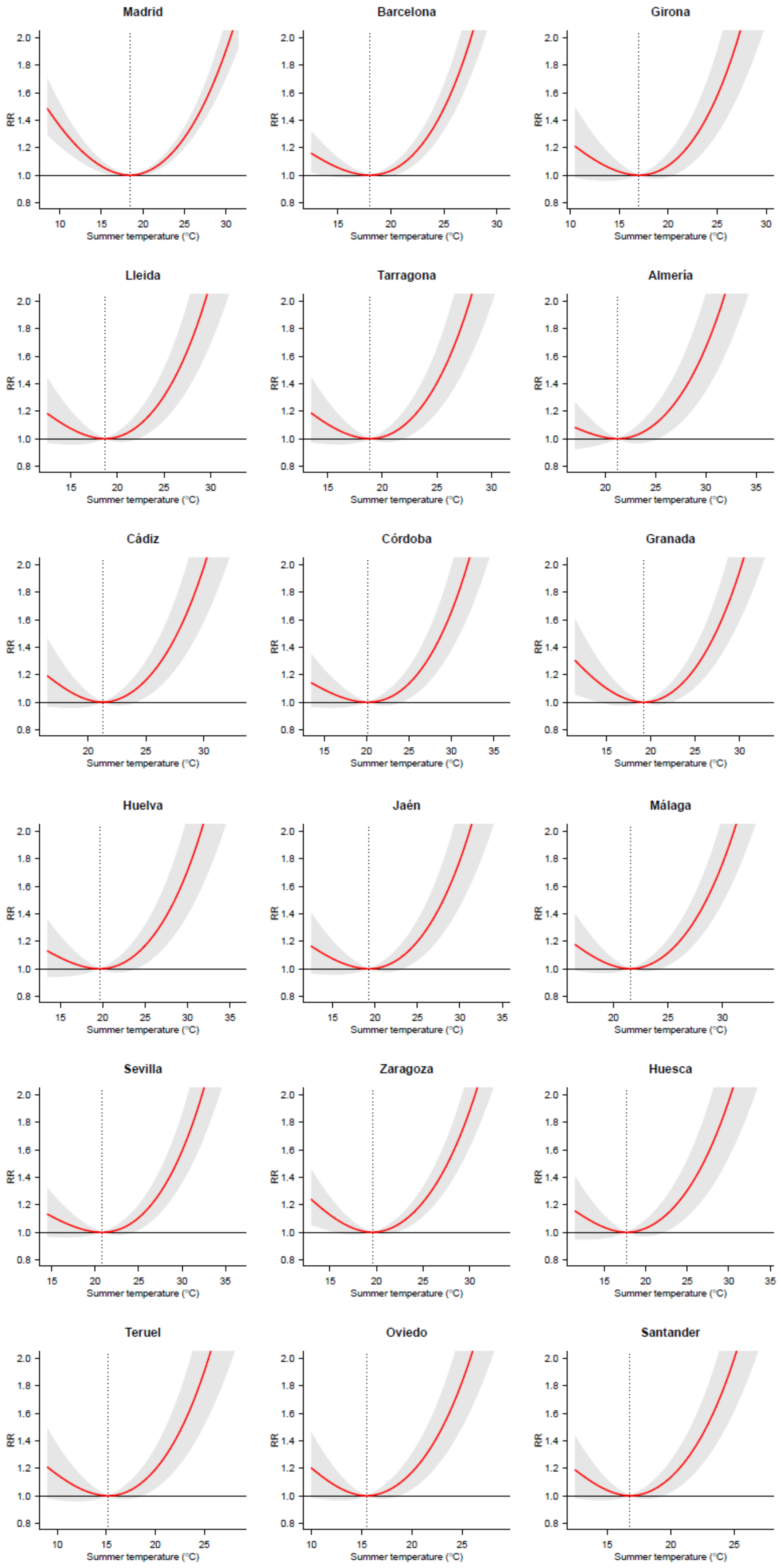

S3 Fig. Temperature-mortality relationships for the 47 provincial capital cities in Spain

Respiratory diseases

Overall

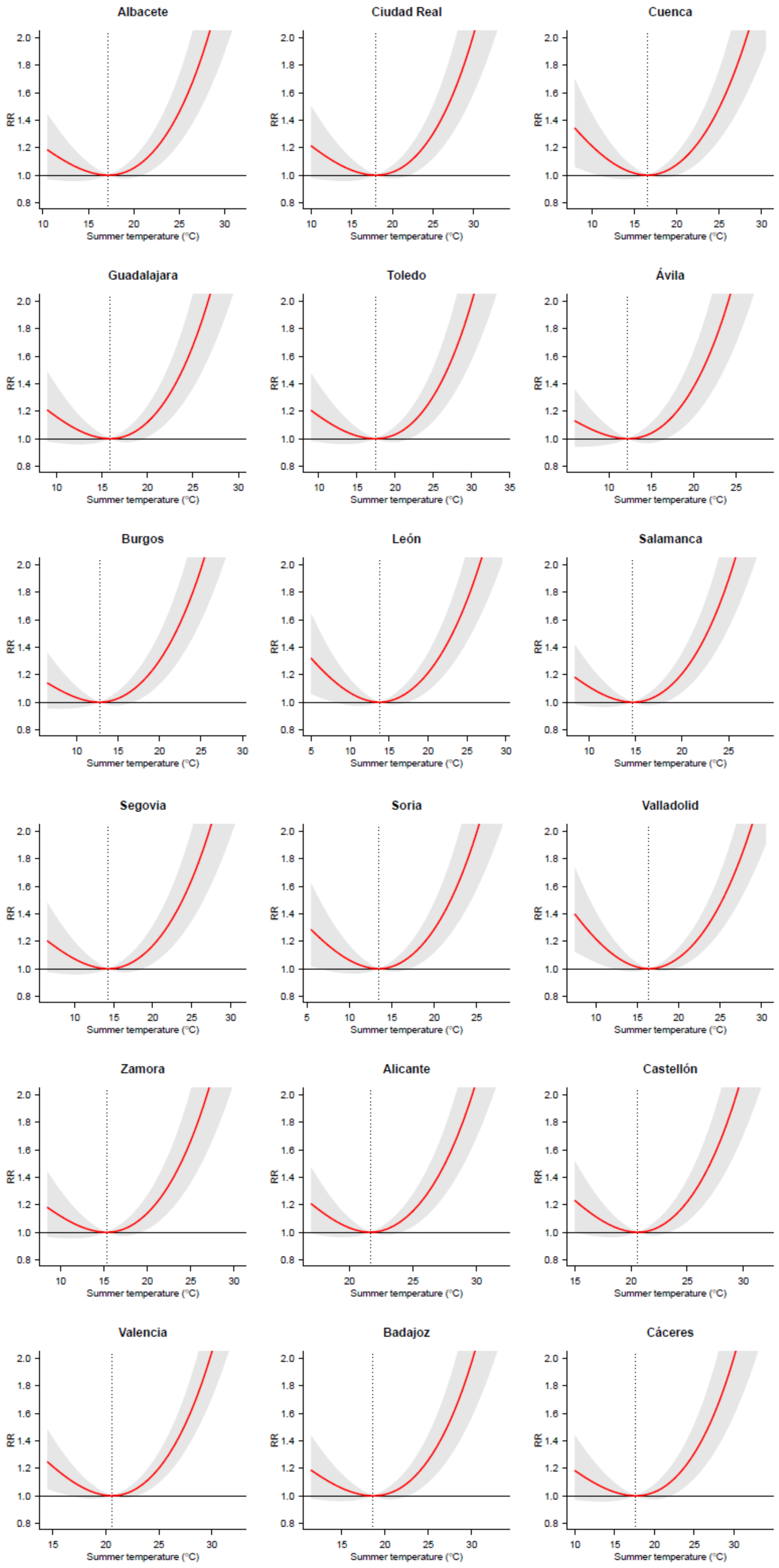

S3 Fig. Temperature-mortality relationships for the 47 provincial capital cities in Spain

Respiratory diseases

Overall

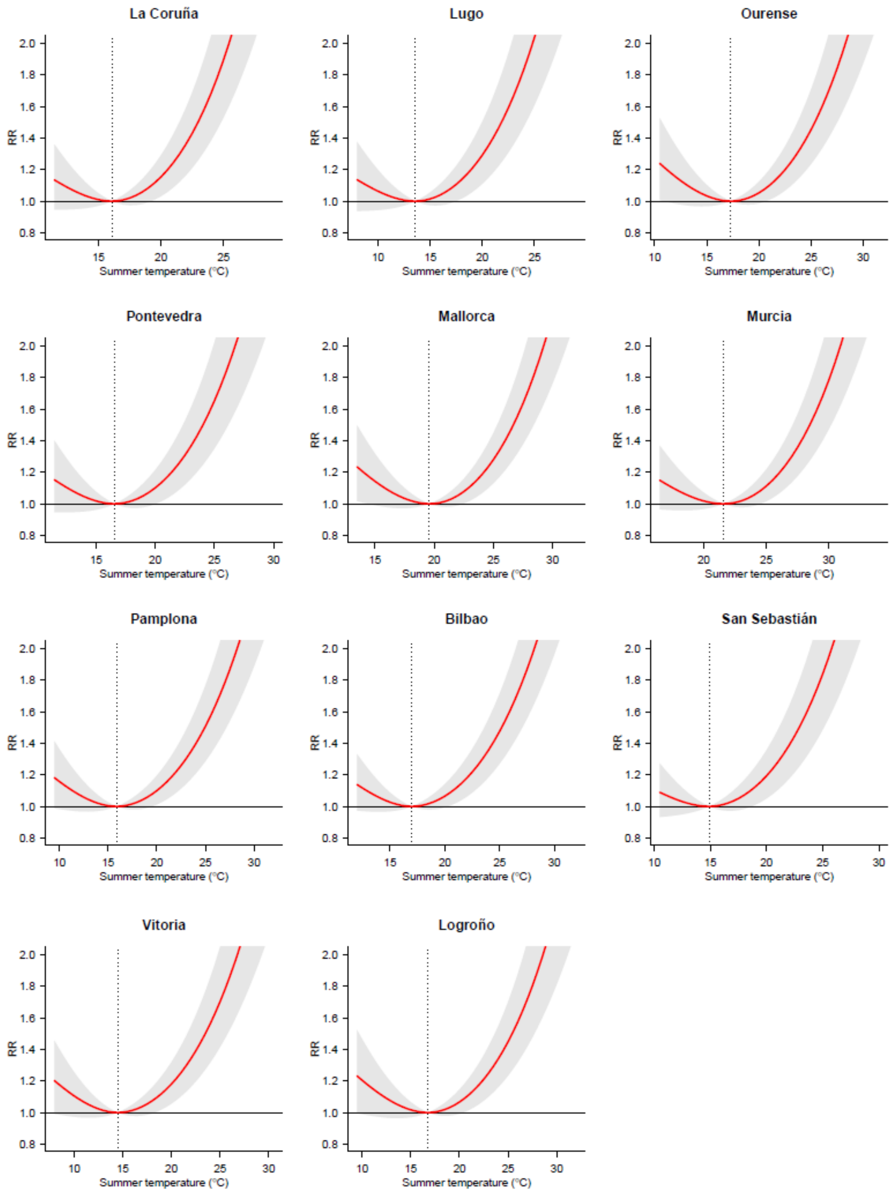

S3 Fig. Temperature-mortality relationships for the 47 provincial capital cities in Spain

Respiratory diseases

Men

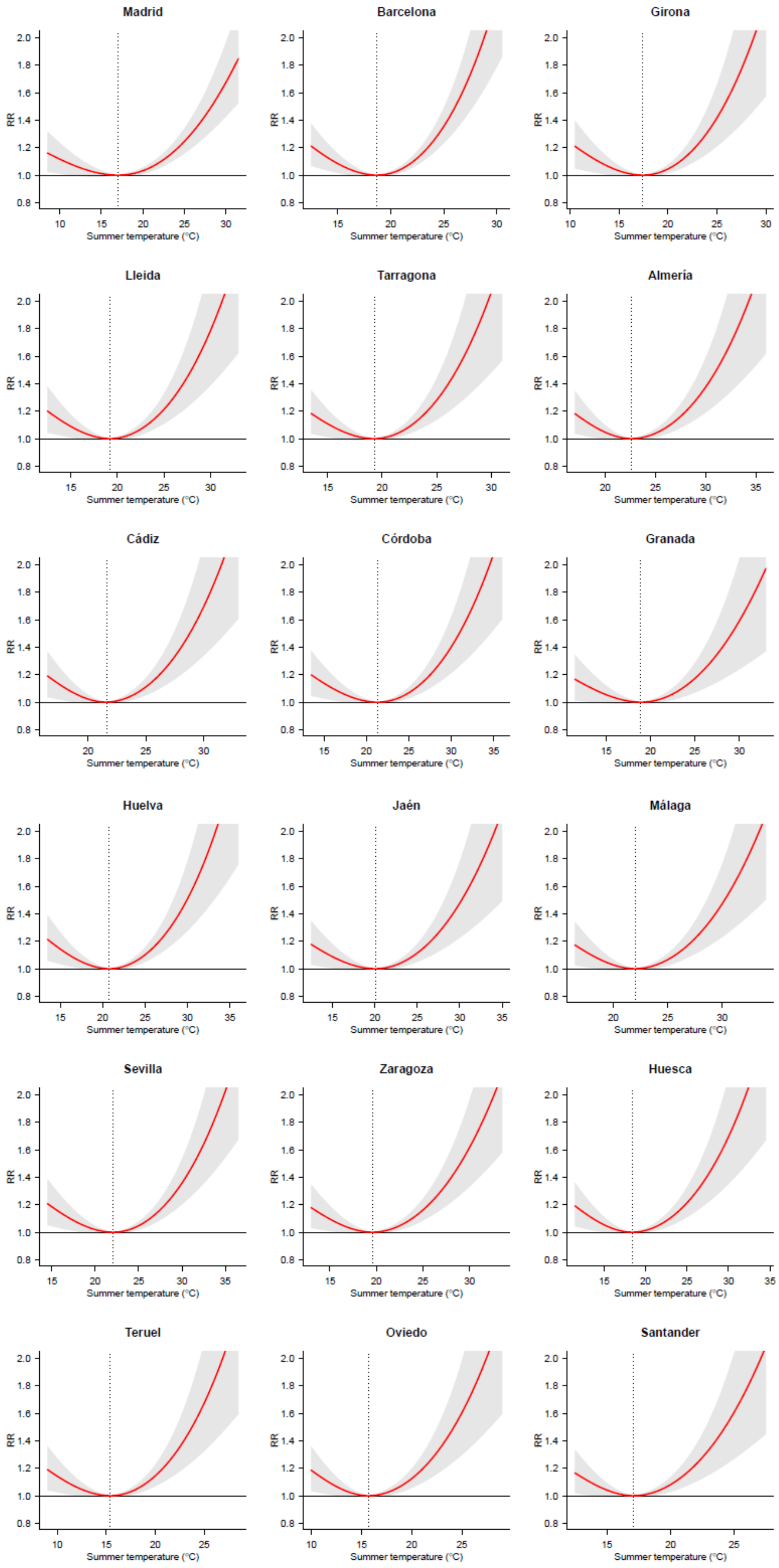

S3 Fig. Temperature-mortality relationships for the 47 provincial capital cities in Spain

Respiratory diseases

Men

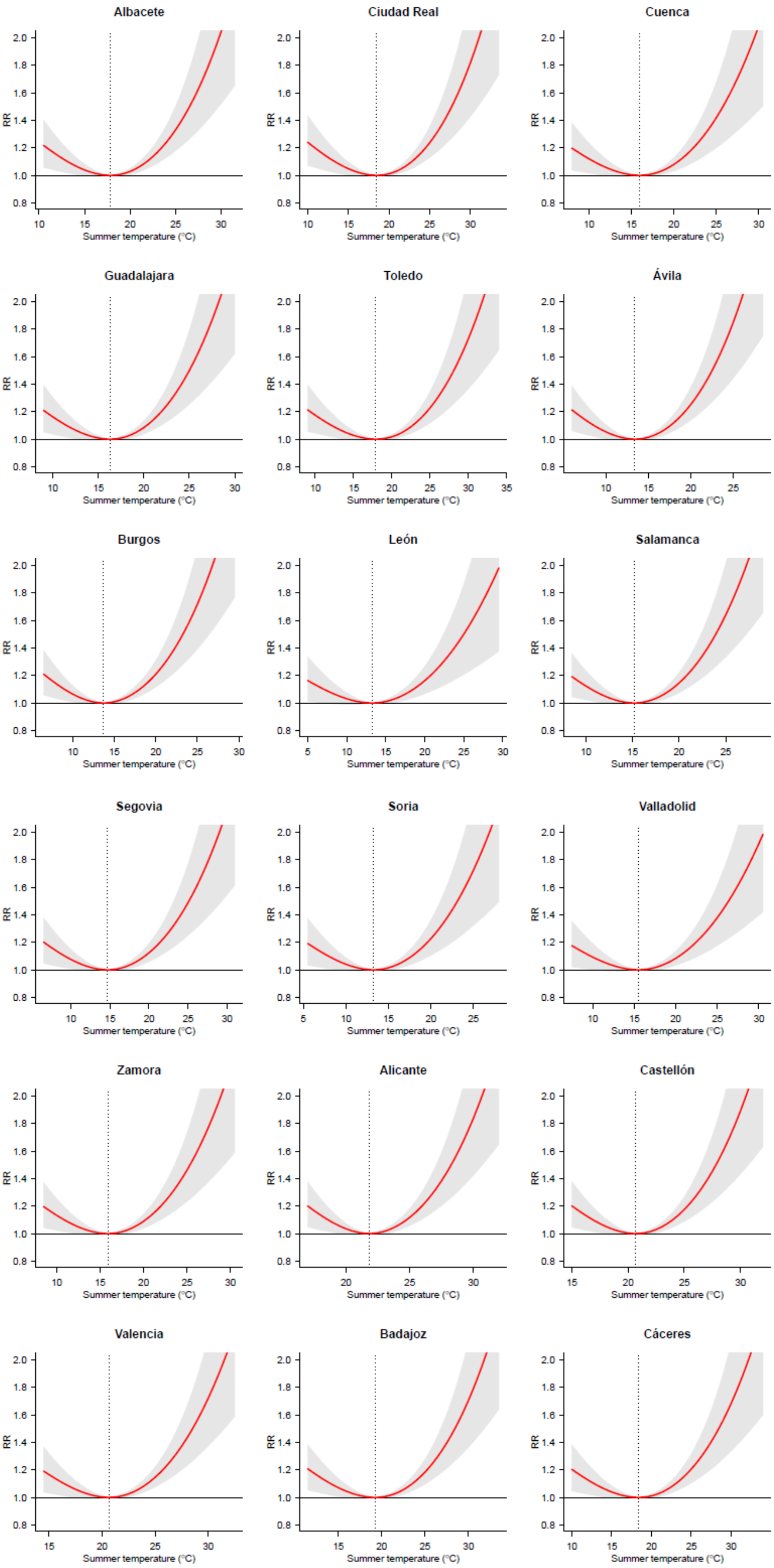

S3 Fig. Temperature-mortality relationships for the 47 provincial capital cities in Spain

Respiratory diseases

Men

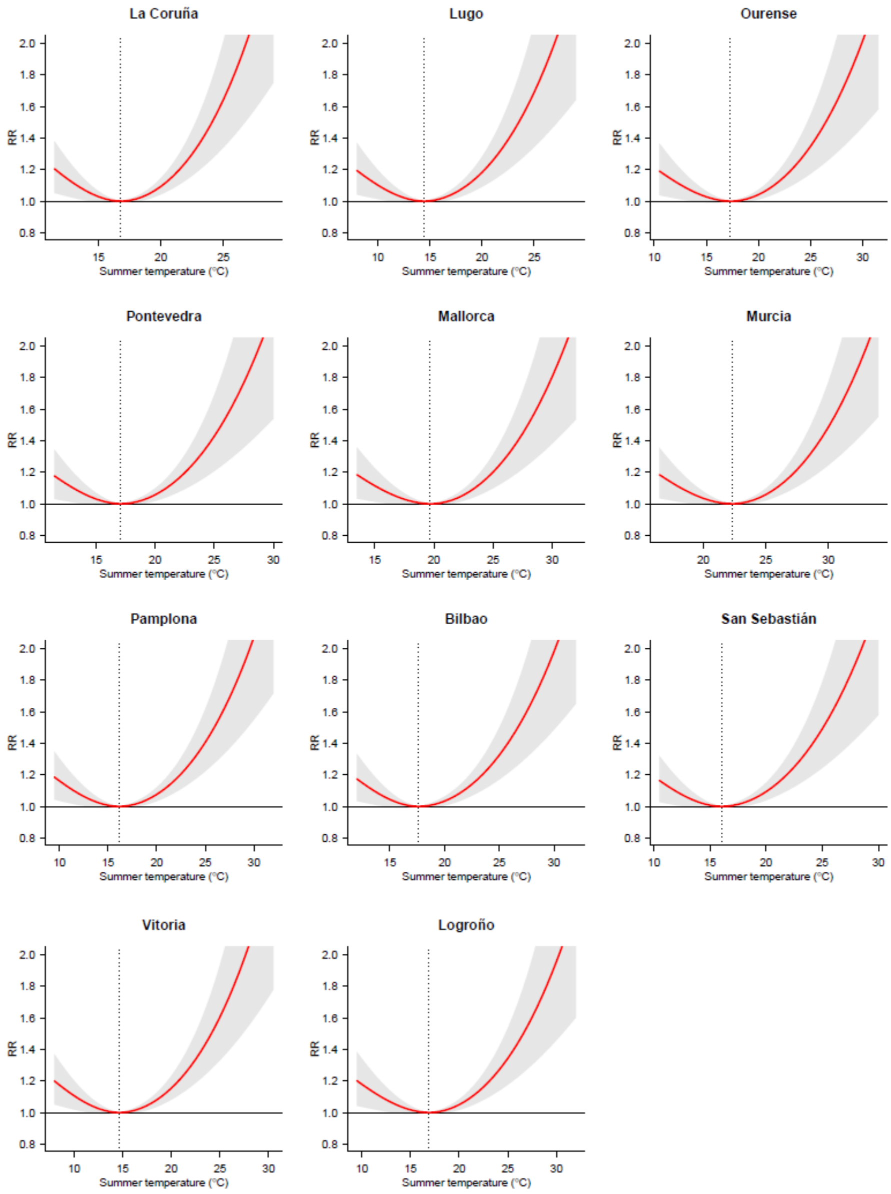

S3 Fig. Temperature-mortality relationships for the 47 provincial capital cities in Spain

Respiratory diseases

Women

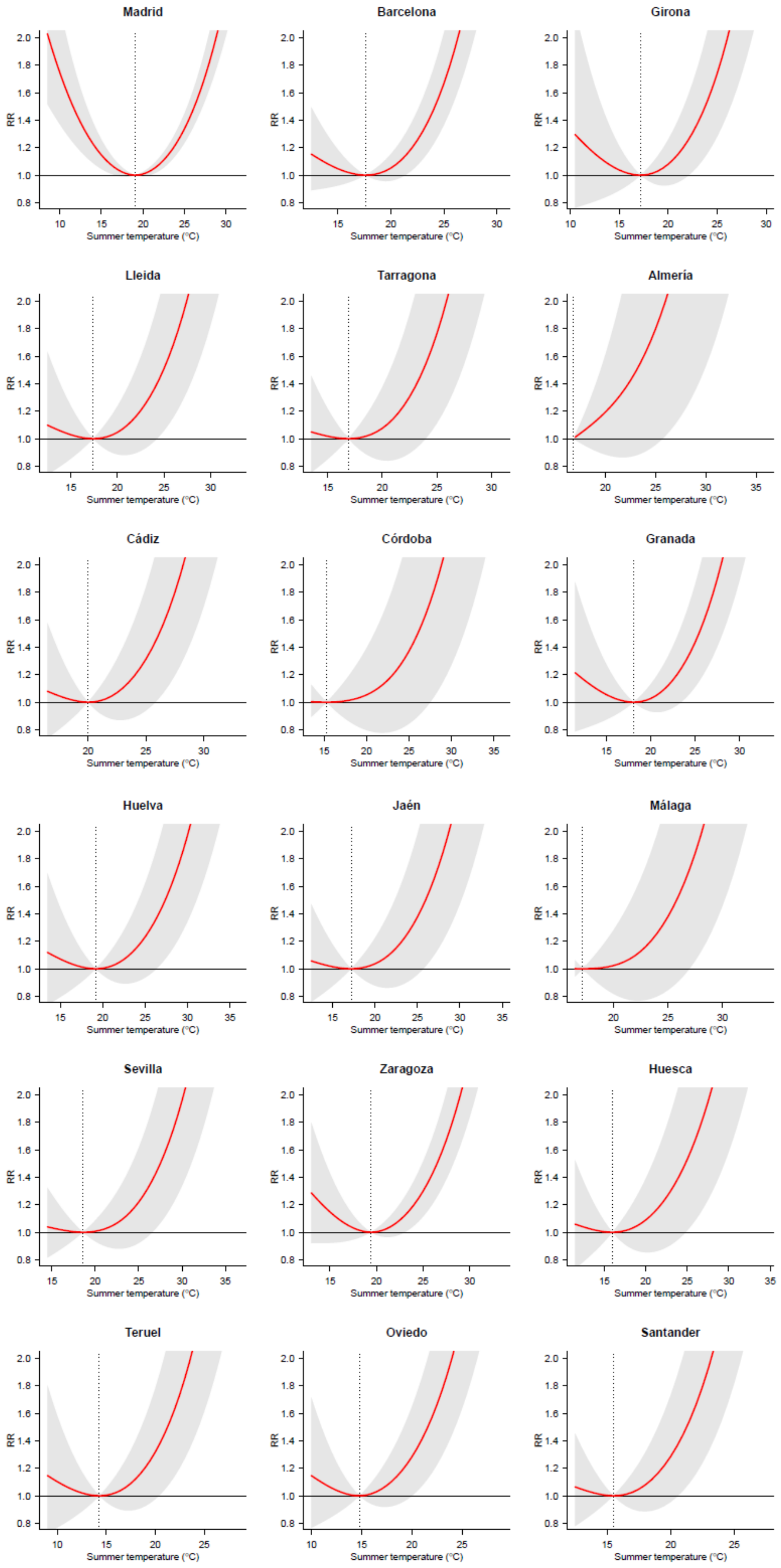

S3 Fig. Temperature-mortality relationships for the 47 provincial capital cities in Spain

Respiratory diseases

Women

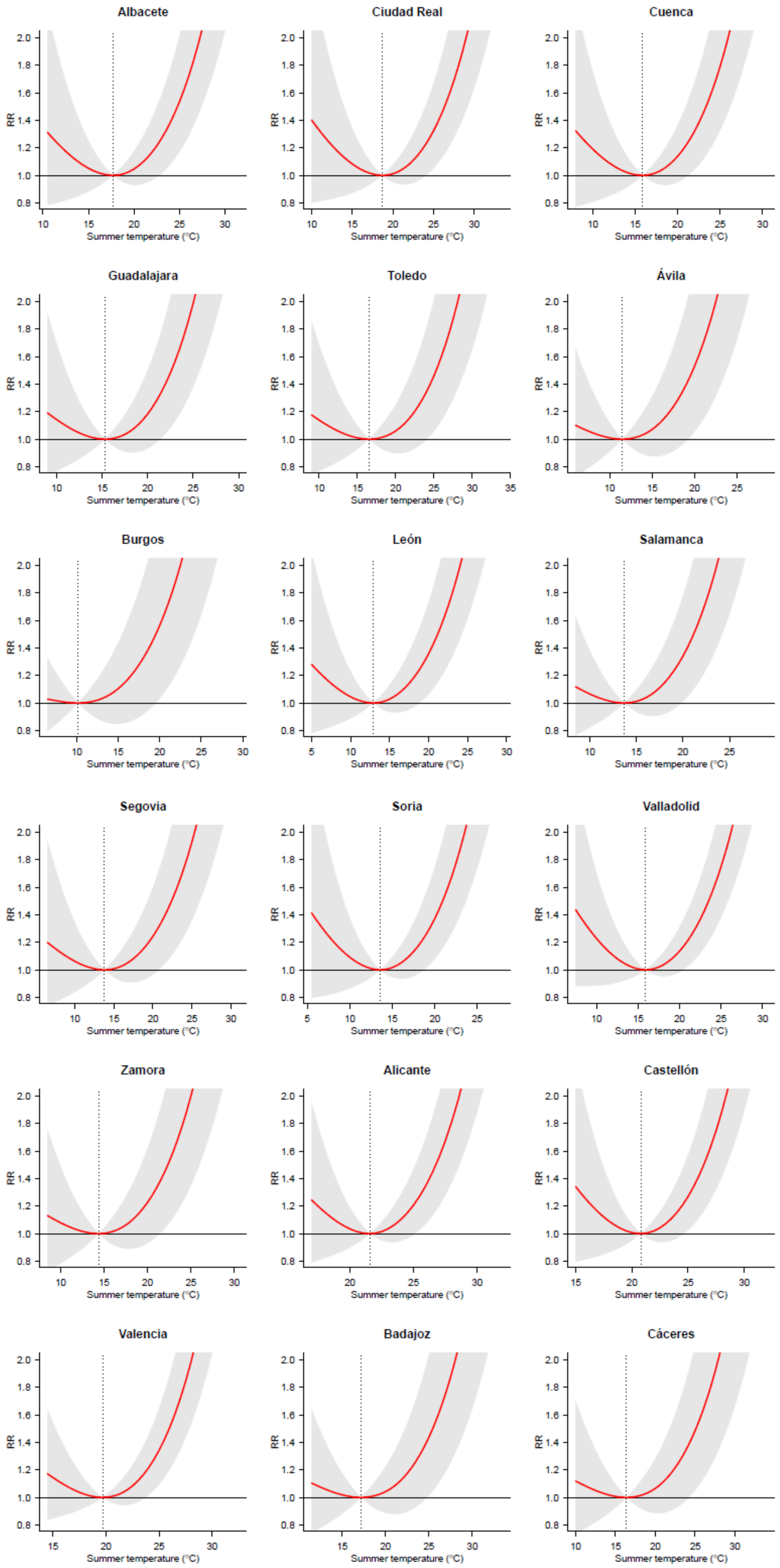

S3 Fig. Temperature-mortality relationships for the 47 provincial capital cities in Spain

Respiratory diseases

Women

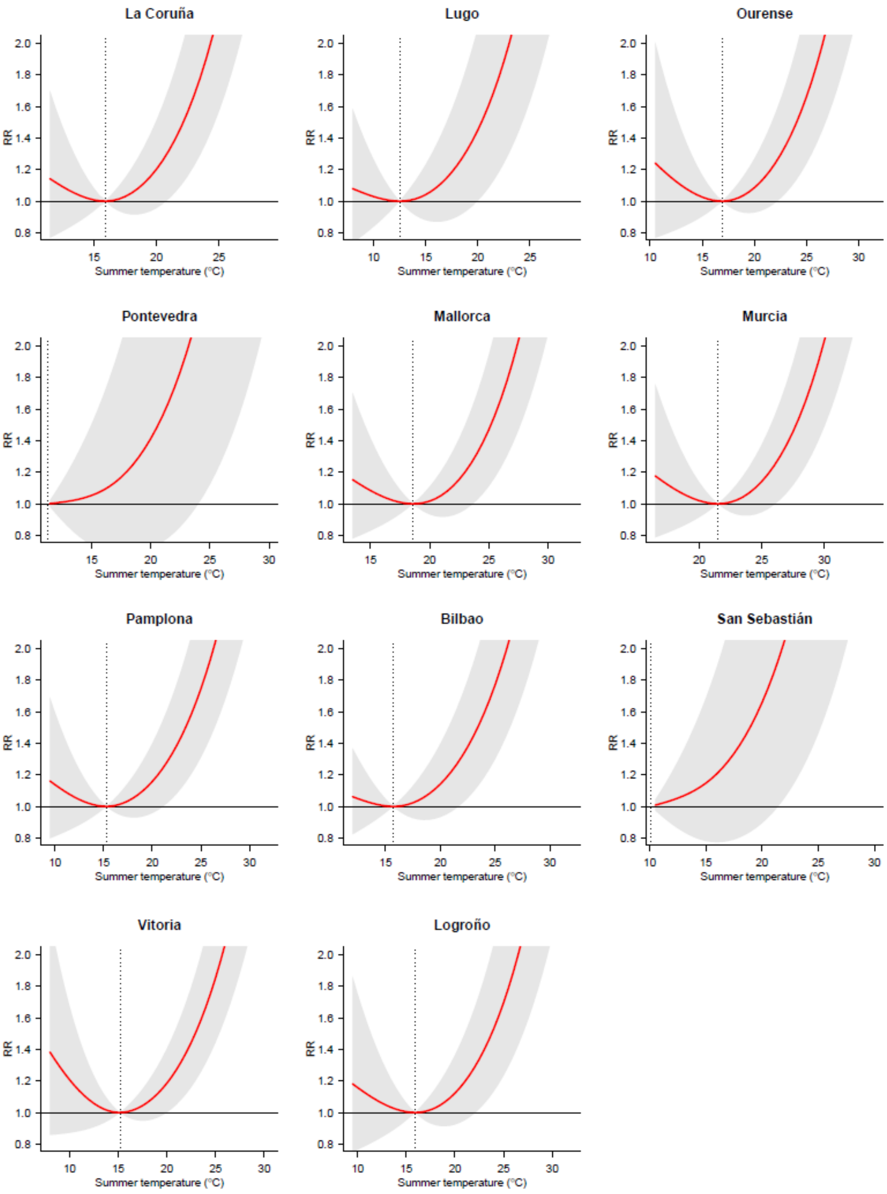

Supplement: S3 Fig — (PDF) [file pmed.1002617.s004.pdf]
