# Supplementary material for: Heat-related mortality trends under recent climate warming in Spain: A 36-year observational study
Source: PLoS Med. 2018 Jul 24;15(7):e1002617. doi: 10.1371/journal.pmed.1002617 (PMC6057624; doi:10.1371/journal.pmed.1002617)

S4 Fig. Estimated minimum mortality temperature for the 47 provincial capital cities in Spain. MMT indicates minimum mortality temperature. Cities are sorted by population size in 2016.

A. Circulatory disease

Men

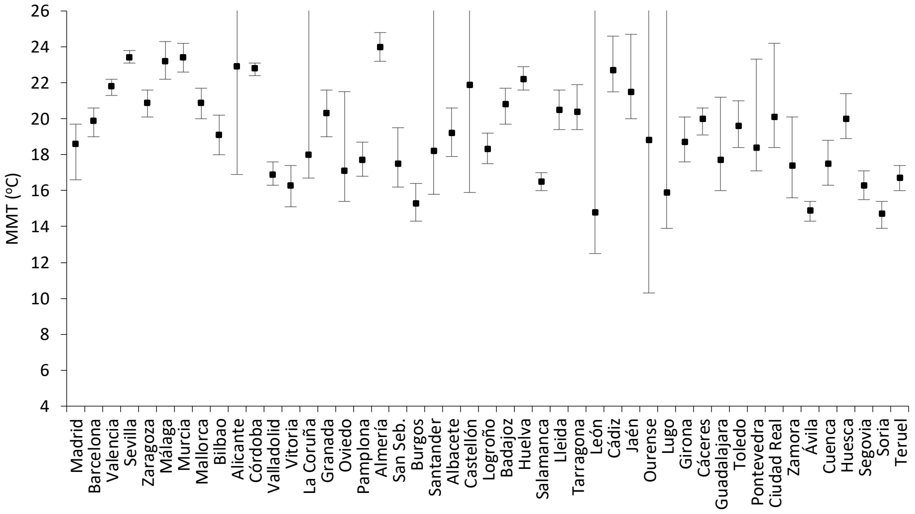

Women

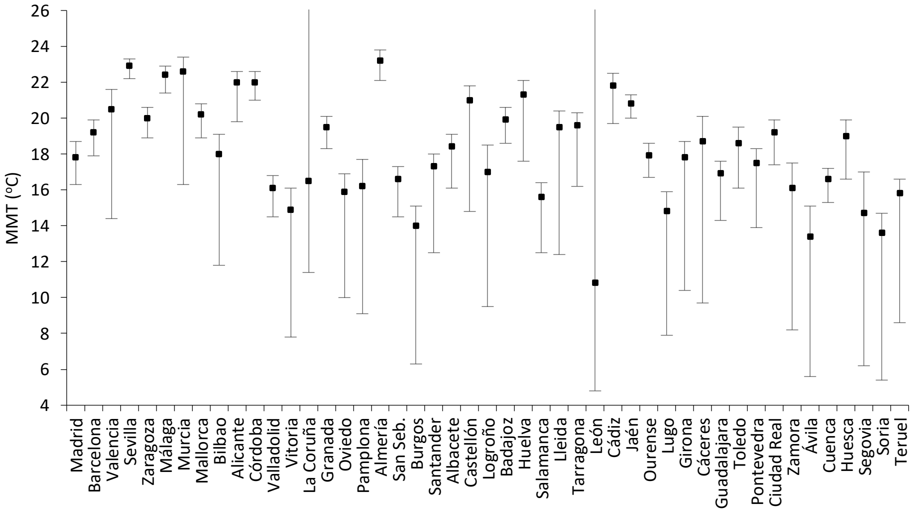

Total

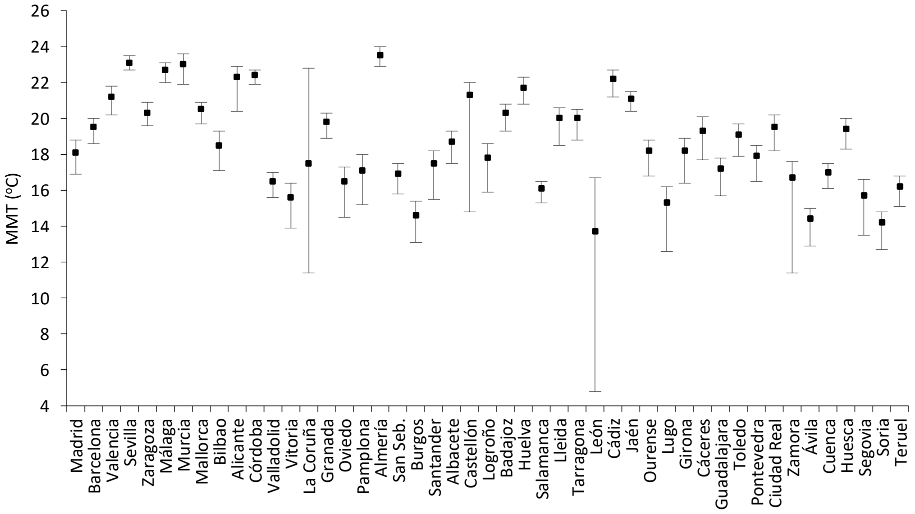

B. Respiratory disease

Men

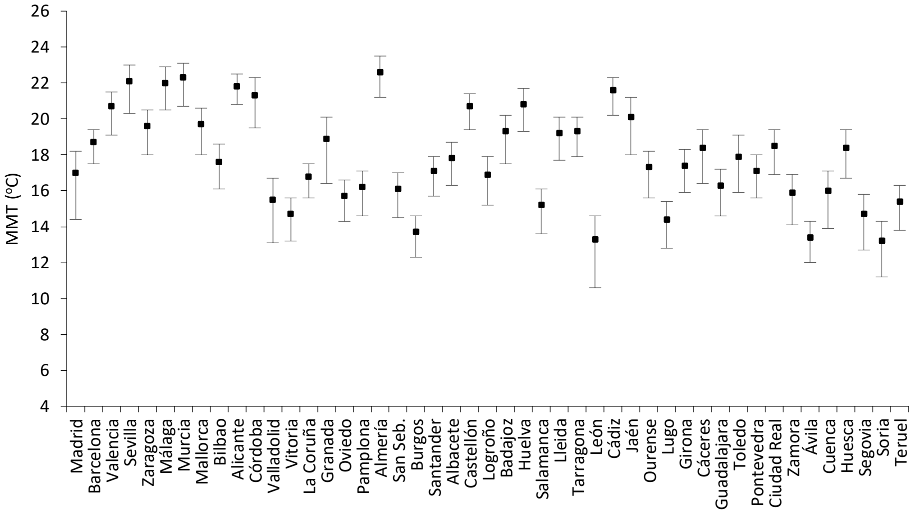

Women

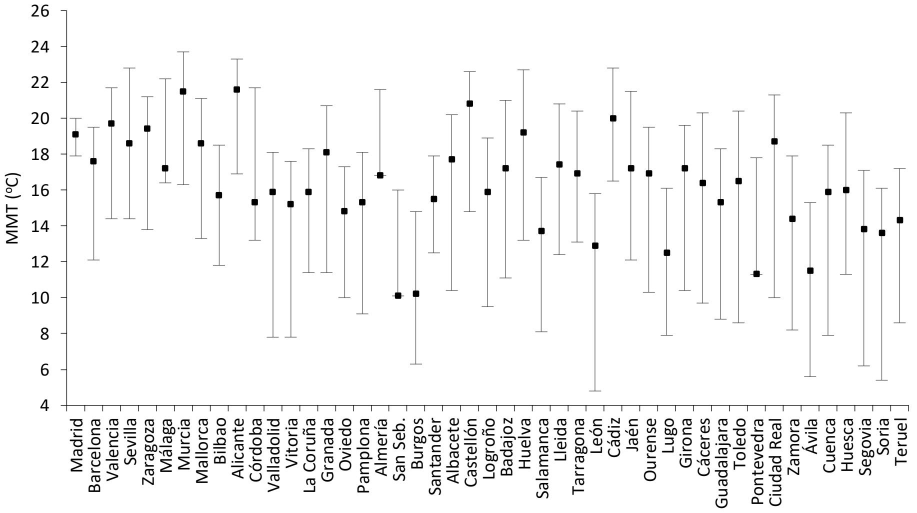

Total

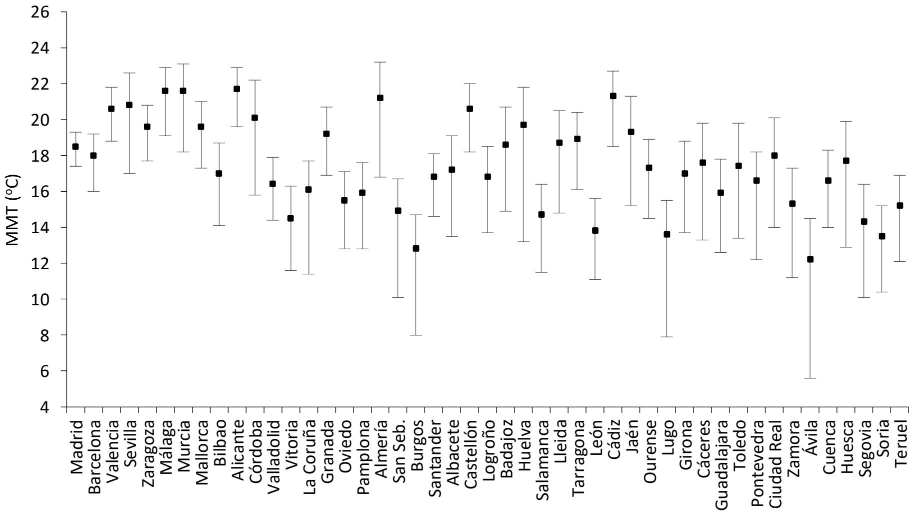

C. Circulatory and respiratory diseases

Men

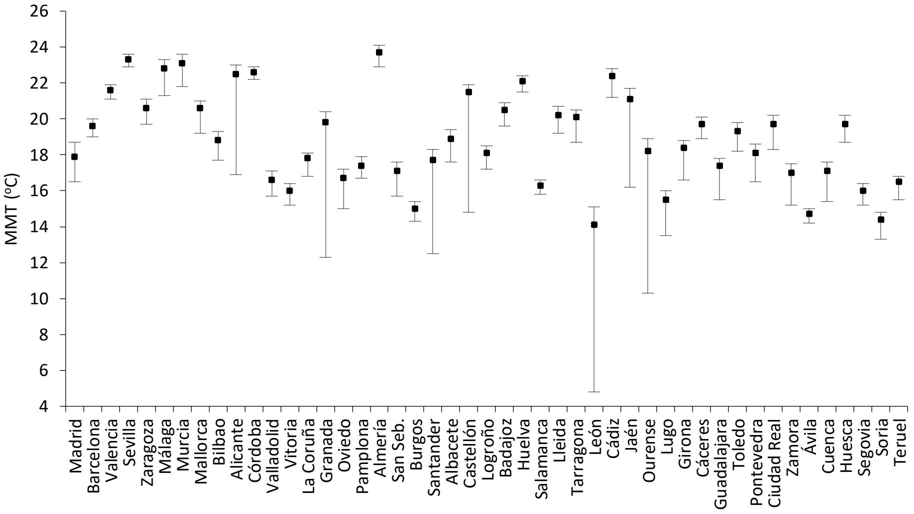

Women

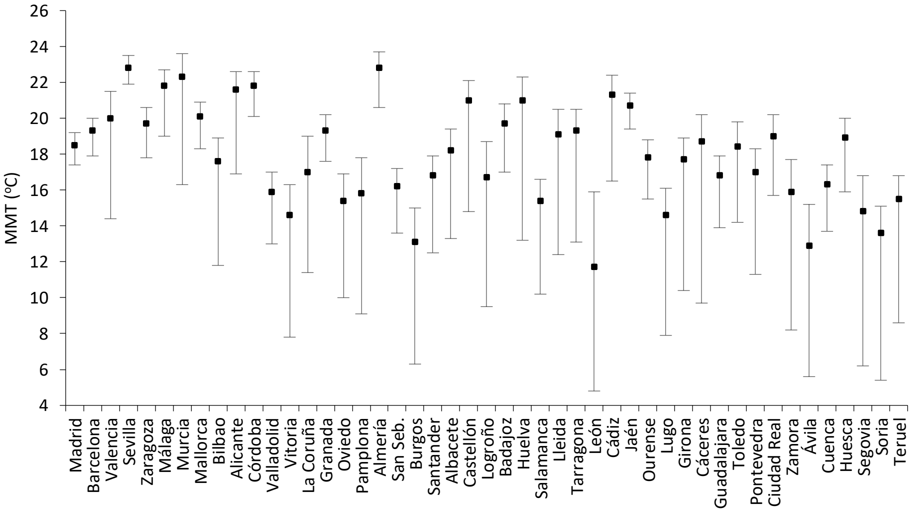

Total

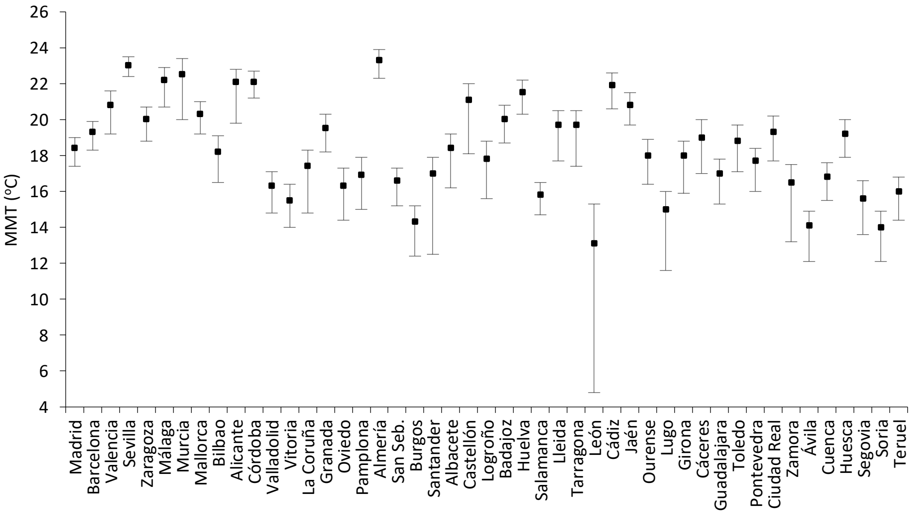

Supplement: S4 Fig — (PDF) [file pmed.1002617.s005.pdf]
