# Supplementary material for: Heat-related mortality trends under recent climate warming in Spain: A 36-year observational study
Source: PLoS Med. 2018 Jul 24;15(7):e1002617. doi: 10.1371/journal.pmed.1002617 (PMC6057624; doi:10.1371/journal.pmed.1002617)

S5 Fig. Relationship for the ensemble of cities between MMT and summer mean temperature. MMT, minimum mortality temperature.

A. Circulatory disease

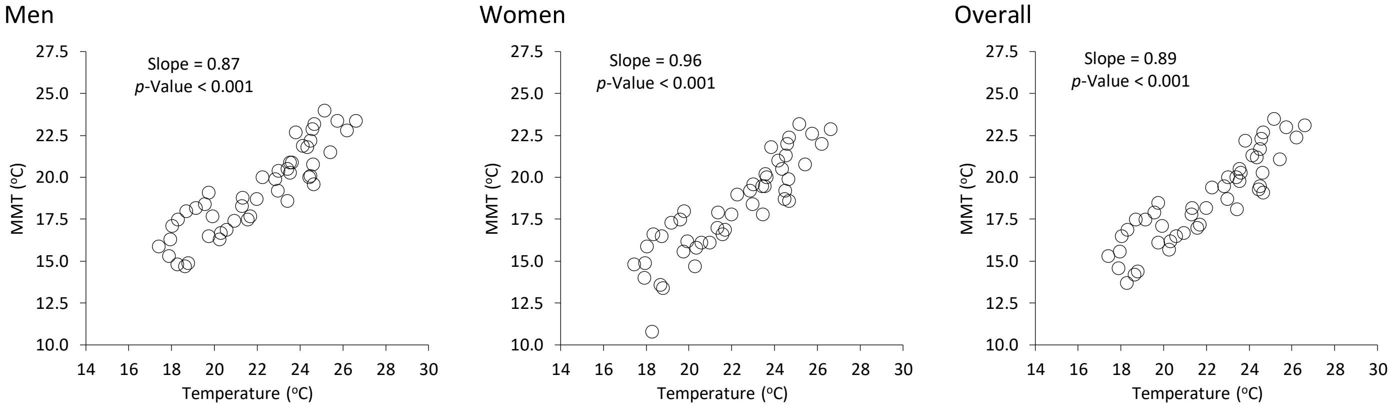

B. Respiratory disease

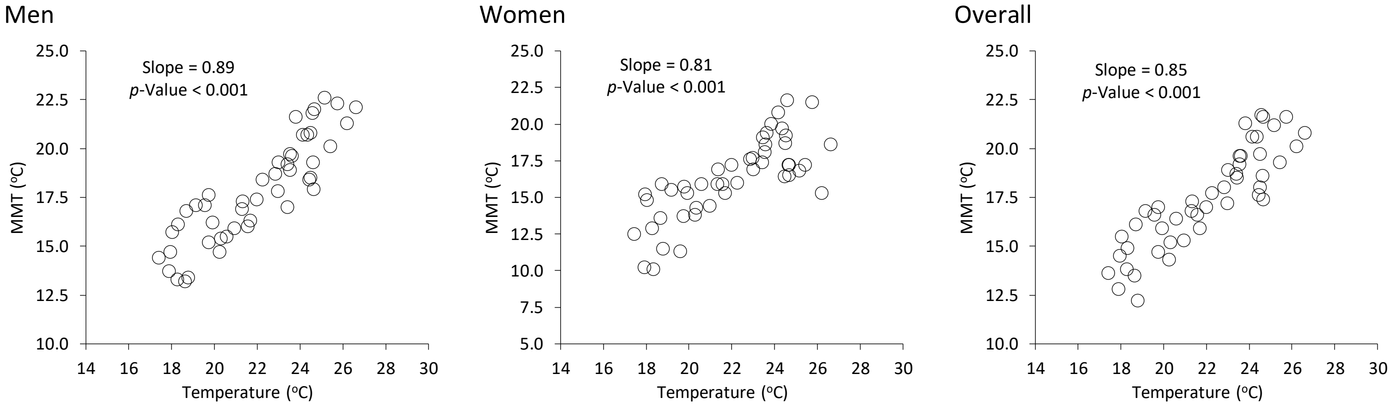

C. Circulatory and respiratory diseases

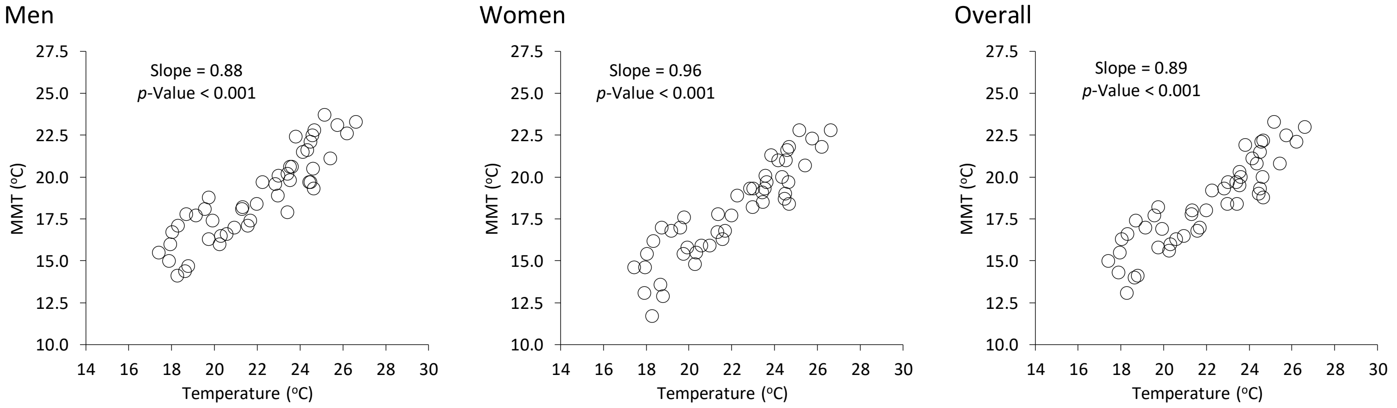

Supplement: S5 Fig — MMT, minimum mortality temperature. (PDF) [file pmed.1002617.s006.pdf]
