# Supplementary material for: Heat-related mortality trends under recent climate warming in Spain: A 36-year observational study
Source: PLoS Med. 2018 Jul 24;15(7):e1002617. doi: 10.1371/journal.pmed.1002617 (PMC6057624; doi:10.1371/journal.pmed.1002617)

S7 Fig. Temporal evolution of the mortality RR at the 90th temperature percentile from the model with interaction (time-varying DLNM). In the left column panels, RR estimates correspond to the 90th temperature percentile of the summer time series for the whole study period, while in the right graphs, they correspond to the 90th temperature percentile of the summer days of the given year (i.e. the 90th percentile of the 122 daily summer values of the year, computed separately for each year). The shaded areas represent the 95% empirical confidence interval. DLNM, distributed lag nonlinear model; RR, relative risk.

A. Circulatory disease

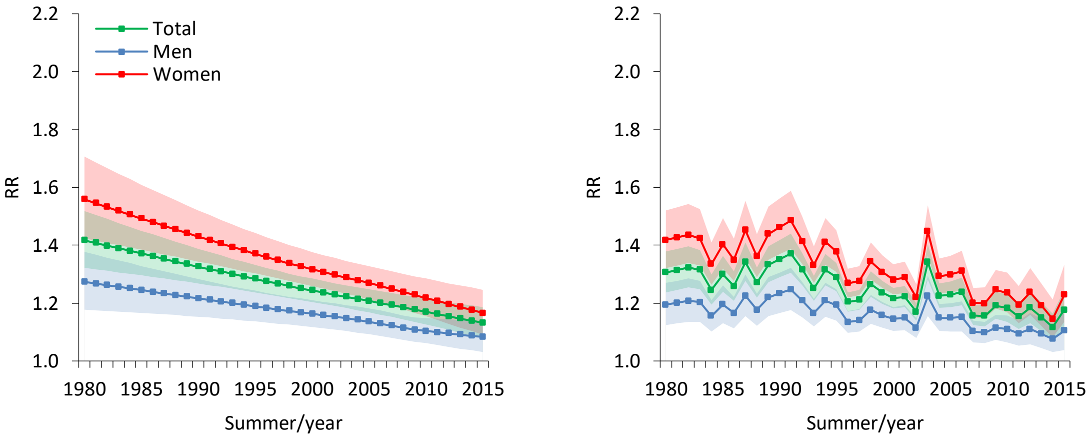

B. Respiratory disease

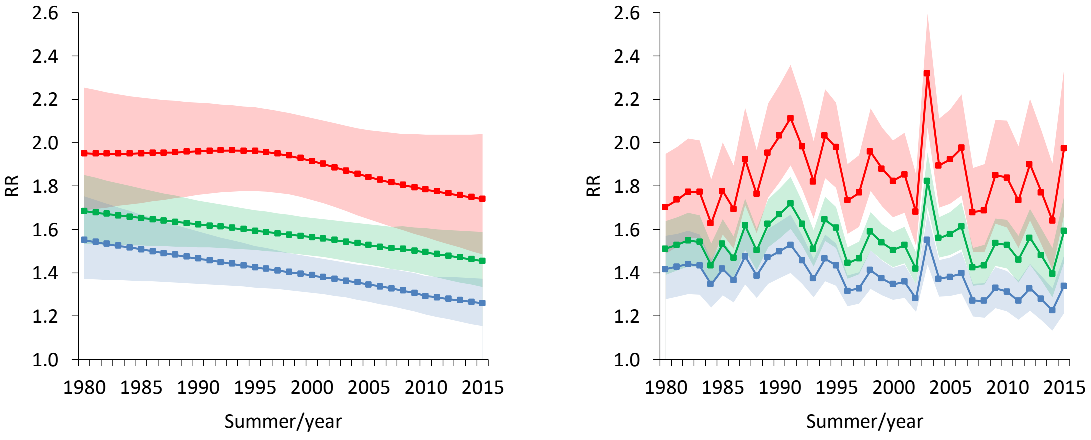

C. Circulatory and respiratory diseases

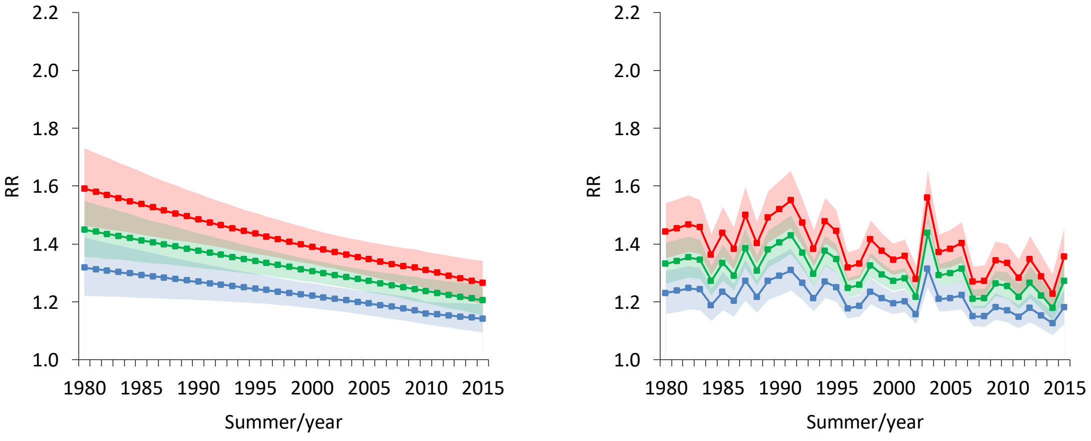

Supplement: S7 Fig — DLNM, distributed lag nonlinear model; RR, relative risk. (PDF) [file pmed.1002617.s008.pdf]
