# Supplementary material for: Heat-related mortality trends under recent climate warming in Spain: A 36-year observational study
Source: PLoS Med. 2018 Jul 24;15(7):e1002617. doi: 10.1371/journal.pmed.1002617 (PMC6057624; doi:10.1371/journal.pmed.1002617)

S8 Fig. Temporal evolution of mortality attributable to moderate and extreme heat for 1980-2015

A. Circulatory disease

Men | Women | Overall

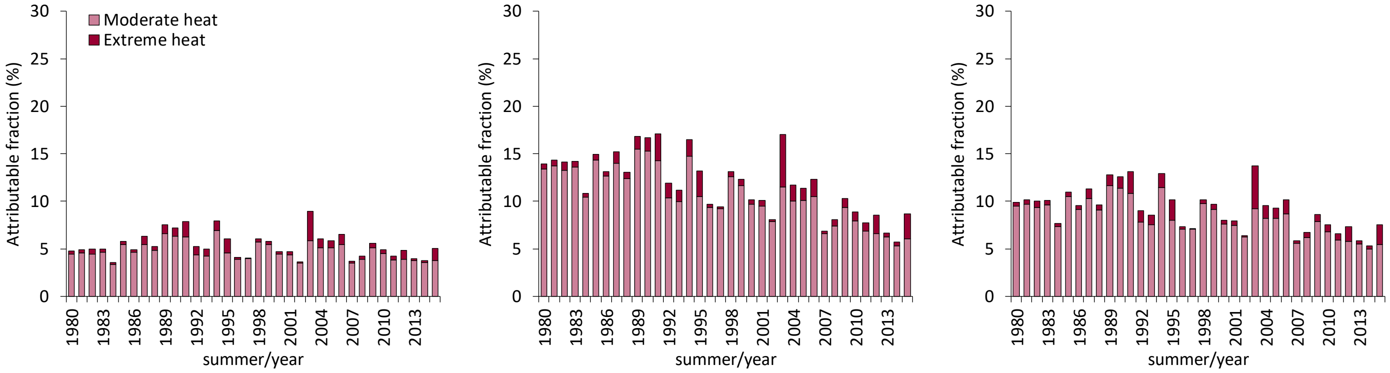

B. Respiratory disease

Men | Women | Overall

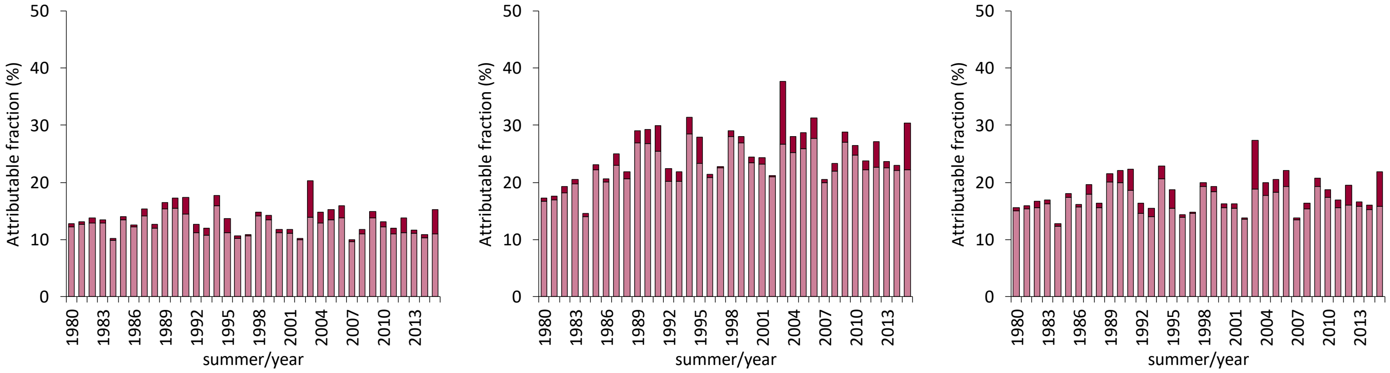

C. Circulatory and respiratory diseases

Men | Women | Overall

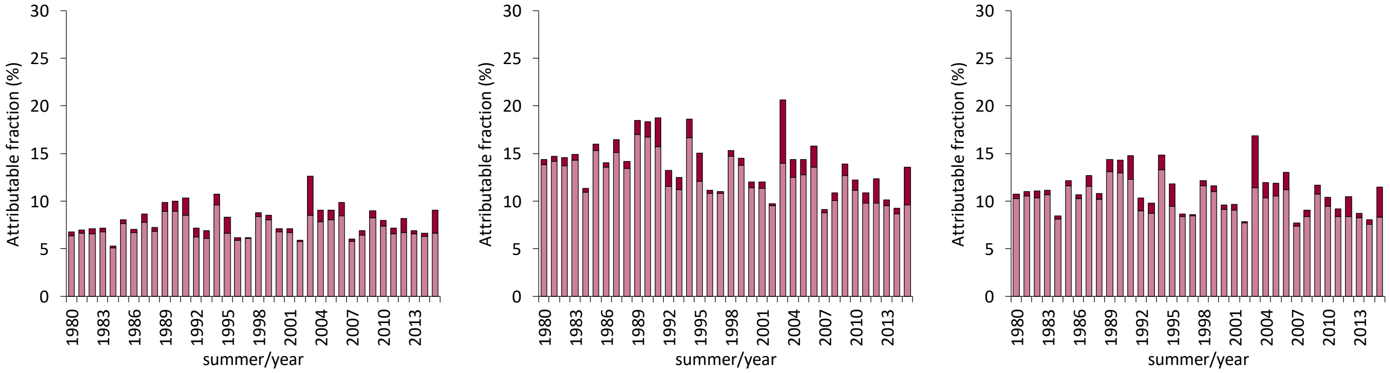

Supplement: S8 Fig — (PDF) [file pmed.1002617.s009.pdf]
