# Supplementary material for: Heat-related mortality trends under recent climate warming in Spain: A 36-year observational study
Source: PLoS Med. 2018 Jul 24;15(7):e1002617. doi: 10.1371/journal.pmed.1002617 (PMC6057624; doi:10.1371/journal.pmed.1002617)
Supplement: S1 Analysis Plan — (PDF) [file pmed.1002617.s010.pdf]

S1 Text. Development of the Statistical Analysis Plan

1. Background

Anthropogenic greenhouse gas emissions have increased summer temperatures in Spain by nearly one degree Celsius on average between 1980 and 2015. However, little is known about the extent to which the association between heat and mortality has been modified. The main objective of the study is to investigate whether the observed warming has been associated with an upward trend in excess mortality attributable to heat or, on the contrary, a decrease in the vulnerability to heat has contributed to a reduction of the mortality burden.

2. Data sources

The Spanish National Statistics Institute (INE) provided daily death counts from circulatory (ICD-9: 390-459, ICD10: I00-I99) and respiratory (ICD-9: 460-519, ICD-10: J00-J99) diseases disaggregated by sex, and covering the period from 1 January 1980 to 31 December 2015 in 47 major Spanish cities. Daily mortality data had no missing value. We derived daily mean 2-meter temperature observations from the European Climate Assessment and Dataset (ECA&D), which were computed as the average between daily maximum and minimum values from meteorological stations. The temperature time series had 1% of missing data.

3. Study population and sample size

The study analysed data from 47 major cities representing about 32% of the total Spanish population, which included 544,491 summer deaths (June-September) corresponding to the period 1980-2015. Circulatory counts represented 78.9% of the total cardiorespiratory mortality, while respiratory deaths accounted for the remaining 21.1%.

4. Variables

The model had daily number of deaths (cause-specific and cause-sex mortality) as the outcome variable and daily mean 2-meter temperature as the exposure variable.

5. Statistical analysis prior to getting comments from peer-reviewers

Temperatures were initially calculated as the mid-year population-weighted average of the daily mean temperature observed in each of the cities included in the analysis, and deaths counts aggregated across cities. We applied standard quasi-Poisson regression models for time-series data, controlling for seasonality and long-term trends, and estimated the temporal variation in heat-related mortality with time-varying distributed lag nonlinear models (time-varying DLNM). The Poisson regression model was given as follows:

$$\text{Log E (Yt)} = \text{intercept} + \text{cb} + \text{dow} + \text{S1(dos, df = 4):factor(year)} + \text{S2(time, df = 1 * decade)} + \text{int(time*cb)}$$

Where *Yt* denotes the series of daily mortality counts; *cb* the cross-basis matrix produced by DLNM; *dow* the day of the week; *S1* the natural cubic B-spline of the day of the season; *S2* the natural cubic B-spline of time; and *int* a linear interaction between the cross-basis and time variables.

6. Changes to the statistical analysis after getting comment from reviewers

Based on comments and suggestions from the reviewers, we performed the analysis in two stages: the regression model is first applied individually in each of the 47 cities in order to estimate location-specific temperature-mortality associations (as described in Section 5), which were then pooled through a random effect meta-analysis.
