# Supplementary material for: Heat-related mortality trends under recent climate warming in Spain: A 36-year observational study
Source: PLoS Med. 2018 Jul 24;15(7):e1002617. doi: 10.1371/journal.pmed.1002617 (PMC6057624; doi:10.1371/journal.pmed.1002617)
Supplement: S1 Table — RR, relative risk. (PDF) [file pmed.1002617.s011.pdf]

S1 Table. Results of the multivariate Wald test for mortality RR curves. RR, relative risk.

		1980 vs. 2015	1985 vs. 20115	1990 vs. 2015	1995 vs. 2015	2000 vs. 2015
Circulatory disease		**< 0.001**	**< 0.001**	**< 0.001**	**< 0.001**	**0.003**
	Men	**< 0.001**	**< 0.001**	**< 0.001**	**0.002**	**0.018**
	Women	**< 0.001**	**< 0.001**	**< 0.001**	**< 0.001**	**0.012**
Respiratory disease		**0.007**	**0.009**	**0.010**	**0.027**	0.099
	Men	**0.017**	**0.019**	**0.026**	**0.045**	0.103
	Women	0.129	0.155	0.185	0.245	0.410
Circ. and resp. diseases		**< 0.001**	**< 0.001**	**< 0.001**	**< 0.001**	**0.008**
	Men	**< 0.001**	**< 0.001**	**< 0.001**	**0.002**	**0.012**
	Women	**< 0.001**	**< 0.001**	**< 0.001**	**0.004**	**0.034**
